# Crystal Structure of Thrombin in Complex with S-Variegin: Insights of a Novel Mechanism of Inhibition and Design of Tunable Thrombin Inhibitors

**DOI:** 10.1371/journal.pone.0026367

**Published:** 2011-10-28

**Authors:** Cho Yeow Koh, Sundramurthy Kumar, Maria Kazimirova, Patricia A. Nuttall, Uvaraj P. Radhakrishnan, Seongcheol Kim, Pudur Jagadeeswaran, Takayuki Imamura, Jun Mizuguchi, Sadaaki Iwanaga, Kunchithapadam Swaminathan, R. Manjunatha Kini

**Affiliations:** 1 Department of Biological Sciences, National University of Singapore, Singapore, Singapore; 2 Institute of Zoology, Slovak Academy of Sciences, Bratislava, Slovakia; 3 NERC Centre for Ecology and Hydrology, Oxford, United Kingdom; 4 Department of Biological Sciences, University of North Texas, Denton, Texas, United States of America; 5 The Chemo-Sero-Therapeutic Research Institute, Kumamoto, Japan; 6 Department of Biochemistry and Molecular Biophysics, Medical College of Virginia, Virginia Commonwealth University, Richmond, Virginia, United States of America; Aligarh Muslim University, India

## Abstract

The inhibition of thrombin is one of the important treatments of pathological blood clot formation. Variegin, isolated from the tropical bont tick, is a novel molecule exhibiting a unique ‘two-modes’ inhibitory property on thrombin active site (competitive before cleavage, noncompetitive after cleavage). For the better understanding of its function, we have determined the crystal structure of the human α-thrombin:synthetic-variegin complex at 2.4 Å resolution. The structure reveals a new mechanism of thrombin inhibition by disrupting the charge relay system. Based on the structure, we have designed 17 variegin variants, differing in potency, kinetics and mechanism of inhibition. The most active variant is about 70 times more potent than the FDA-approved peptidic thrombin inhibitor, hirulog-1/bivalirudin. *In vivo* antithrombotic effects of the variegin variants correlate well with their *in vitro* affinities for thrombin. Our results encourage that variegin and the variants show strong potential for the development of tunable anticoagulants.

## Introduction

**Figure 1:**
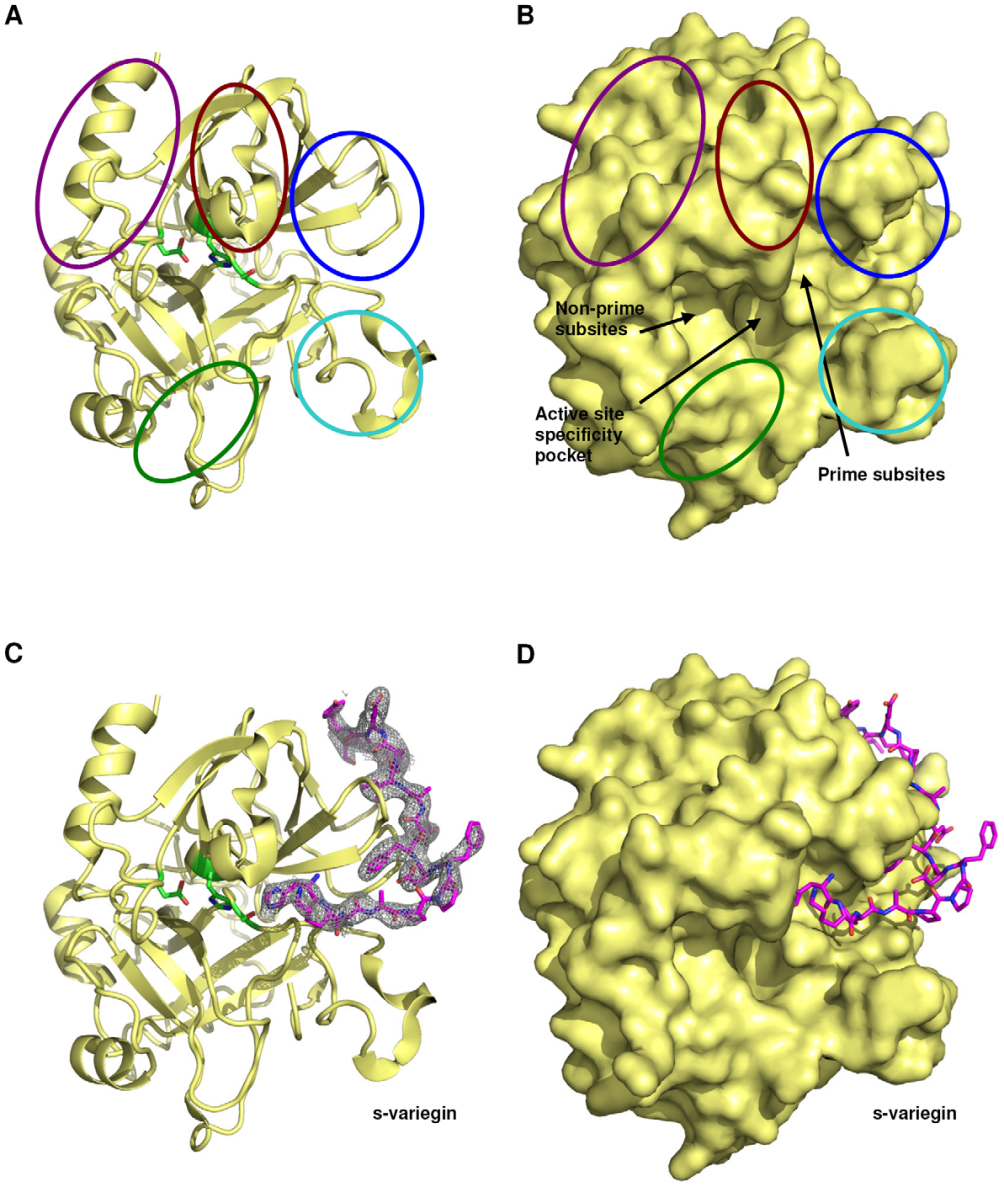
Structure of thrombin:s-variegin complex. (**A**) Thrombin (yellow) shown in the classical orientation in ribbon (without s-variegin). Side chains of catalytic triad, ^T^Asp102, ^T^His57 and ^T^Ser195 are shown in sticks (green). The 60-loop, autolysis loop and Na^+^-binding loop are circled in brown, cyan and green, respectively. Parts of thrombin forming the anion-binding exosite-I and exosite-II are circled in blue and purple, respectively. (**B**) Surface representation of thrombin (yellow) in the same orientation as (a). Locations of active site specificity pocket, non-prime and prime subsites are indicated by arrows. (**C**) The structure of thrombin (yellow) in the same orientation as above shown in complex with s-variegin (pink) together with its electron density map (2Fo-Fc) shown contoured at 0.9σ. (**D**) Surface representation of thrombin in complex with s-variegin (pink).

Serine proteinases in the blood coagulation cascade are important molecules in maintaining the integrity of hemostasis. Among them, thrombin (factor IIa) plays significant pro- and anti- coagulation roles. The active site contains the classical catalytic triad – His57, Asp102 and Ser195 (Figure 1A). Compared to other blood coagulation serine proteinases, thrombin has a prominent active site cleft, which is deep and narrow. Two insertion loops (called the 60-loop with residues Leu59-Asn62 and the autolysis-loop, residues Leu144-Gly150) form the wall of the cleft (Figure 1A–B) [1], [2]. The thrombin active site surfaces that interact with substrate residues, at N-terminal to the scissile bond, are described as ‘non-prime subsites’ (S subsites). Similarly, the surfaces of the active site which are in contact with substrate residues, at C-terminal to the scissile bond, are described as ‘prime subsites’ (S′ subsites) (Figure 1B).

In addition, exosite-I is the surface near the prime subsites. The bottom of exosite-I is a deep, canyon-like cleft that extends from the prime subsites. The walls of the cleft are formed by two surface loops, Phe34-Leu39 (described as the 34-loop) and Lys70-Glu80 (the 70-loop) [2], [3]. In contrast to the apolar nature of the canyon-like cleft, the surface of exosite-I is dominated by several positively-charged residues [4]. Exosite-II, another surface near the ‘non-prime subsites’, is even more basic (Figure 1B). The occupancy of either exosites can induce allosteric changes to the active site to enhance catalysis. The binding of Na^+^ to the Na^+^ binding loop (Cys220-Trp225) (Figure 1A) favors procoagulant functions of thrombin whereas Na^+^-free thrombin favors anticoagulant functions such as increased protein C activation [5]. The activity and fate of thrombin is directed by competition for its exosites and differences in distribution of its substrates and cofactors [6].

Imbalances in blood coagulation may give rise to either loss of clotting activity, leading to hemorrhagic disorders, or unwanted clot formation, resulting in thrombosis. In particular, thrombosis causes high morbidity and mortality due to vascular occlusion and consequent myocardial infarction, stroke, pulmonary embolism, or deep-vein thrombosis. Increased atherosclerosis and thromboembolic disorders, associated with changing food habits and lifestyles, are increasing the demand for anticoagulant agents [7], [8]. Heparin and warfarin are the cornerstones of anticoagulation therapy. Unfortunately, both classes of drug have well-documented limitations such as a narrow therapeutic window and highly variable dose-response [9]. These limitations drive continual and intense efforts to develop new, efficacious and safe anticoagulants, especially for targeting specific coagulation factors [9]. Thrombin is one of the main targets for inhibition, owing to its pivotal role in coagulation. Several direct thrombin inhibitors, such as hirudin [10], hirulog-1/bivalirudin [11], argatroban [12] and dabigatran [13], are currently available in the market. Among them, hirudin and hirulog-1/bivalirudin are both developed from hematophagous parasites and their success continues to inspire the search for more novel anticoagulants from these sources [14], [15].

Recently, we described variegin, a novel, fast and tight-binding competitive inhibitor of thrombin (refers to the α form of thrombin unless otherwise stated) isolated from the tropical bont tick, *Amblyomma variegatum* [16]. Like hirudin/hirulog, variegin targets the thrombin catalytic site and exosite-I. However, unlike other naturally occurring thrombin inhibitors, variegin interacts with the thrombin prime subsites in addition to exosite-I [17]. Variegin has several potential advantages over hirulog-1/bivalirudin: (i) synthetic variegin (s-variegin) contains only _L_-amino acids, while hirulog-1/bivalirudin has a _D_-Phe [16]; (ii) inhibition by variegin is about 9 times greater than hirulog-1/bivalirudin; (iii) the cleavage product of variegin (MH22) remains tightly bound to thrombin (about 400 fold stronger than cleaved hirulog-1/bivalirudin). Most importantly, MH22 potently and noncompetitively inhibits thrombin whereas cleaved hirulog-1/bivalirudin, paradoxically, activates the function of the thrombin active site [17].

We solved the crystal structure of the thrombin:s-variegin complex at 2.4 Å resolution in order to understand the molecular interactions between thrombin and variegin. Based on the structure and data on thrombin inhibitors, a series of peptides was designed to analyse the structure-function relationships. These peptides cover a diverse spectrum of properties: potency, kinetics, mechanism of inhibition, affinities (ranging from nanomolar to picomolar values) with fast, slow, tight-binding and competitive and noncompetitive inhibition. Finally, *in vivo* activities of selected peptides were examined using a venous thrombosis model involving zebrafish larvae.

## Methods

### Synthesis, purification and mass spectrometry of peptides

Synthesis, purification and mass spectrometry analysis of all peptides were performed as described elsewhere [16]. Peptides were named with two alphabets representing the first two residues in their sequence, followed by a number representing their respective length. Position of point mutants are added after the number and italicized. Modifications to amino acids are indicated by superscript. As sulfate groups are acid labile, the peptides containing sulfotyrosine (DV24*Y^sulf^*, DV24*K10RY^sulf^* and MH18*Y^sulf^*) were cleaved from resins with 90% aqueous trifluoroacetic acid on ice for 5 h.

### X-ray crystallography

Lyophilized recombinant human thrombin [18], [19] and s-variegin were dissolved and mixed in buffer containing 50 mM HEPES (pH 7.4) and 375 mM NaCl to final concentrations of 20 mg/ml and 3 mg/ml (1∶1.5 molar ratio), respectively. Crystallization was achieved using the hanging drop vapor diffusion method. Typically, 1 µl of protein solution was mixed with 1 µl of precipitant buffer (100 mM HEPES buffer pH 7.4, containing 20 to 25% PEG 8000) and equilibrated against 1 ml of the precipitant buffer at 4°C. X-ray diffraction data were collected at Beamline X29 (National Synchrotron Light Source, NY, USA). Prior to data collection, crystals were briefly soaked in a cryoprotectant solution containing the mother liquor, supplemented with 25% (v/v) glycerol. Data sets were collected using the Quantum 4 CCD detector and were processed using HKL2000 [20]. As the χ^2^ values for the P1 unit-cell were better than those for the C2 unit-cell during data integration (around 1.0 against 3.5 and above), data were first processed under the P1 unit-cell orientation and then transformed to the C2 orientation using the transformation A = 2c+a, B = a and C = b, where a,b,c and A,B,C are the P1 and C2 unit-cell vectors, respectively.

The structure of thrombin:s-variegin complex was solved by the molecular replacement method using PHASER [21] at 2.4 Å resolution. The coordinates of the thrombin-hirulog-3 structure (PDB code: 1ABI) [22] were used as a search model. Several cycles of map fitting using program COOT [23] and refinement using program REFMAC5 [24] with one TLS [25] group per chain of thrombin led to convergence of R-values. The crystallographic and refinement statistics are listed in Table 1. The correctness of stereochemistry of the model was verified using PROCHECK [26] and MolProbity [27]. The geometry of the thrombin molecule is comparable to that of other structures at this resolution. The peptide is relatively more flexible. The coordinates of the structure were deposited with the RCSB Protein Data Bank under the entry code 3B23. Online server PISA [28] was used to analyze the protein-peptide interface. Throughout the manuscript, the residues of thrombin and s-variegin are marked with superscripted prefixes ‘T’ and ‘V’, respectively. The chymotrypsinogen numbering system is used for numbering the thrombin residues, as first described here [29].

**Table 1:** Crystallographic data and refinement statistics.

Data set	Thrombin:s-variegin complex	
***Crystal***		
Space Group	P1	C2
Unit Cell Parameters (Å, °)	a = 50.8	A = 124.7
	b = 61.58	B = 50.8
	c = 67.3 Å	C = 61.5 Å
	α = 98.1	AL = 90
	β = 112.2	BE = 98.7
	γ = 89.9°	GA = 90°
***Data collection***		
Resolution range (Å)	50−2.4	
Wavelength (Å)	0.9795	
Total number of reflections	52,825	
Unique reflections	29,154	15,137
Completeness (%)	88.1 (56.8)	98.1 (97.0)
*I/σI*	25.1 (7.0)	20.0 (5.4)
Redundancy	1.9 (1.7)	3.6 (3.1)
R_merge_ (%)	2.4 (8.5)	5.3 (15.4)
***Refinement and quality***		
Resolution range (Å) I>σ(I)		8−2.4
R_work_		0.208
R_free_		0.259
RMSD bond lengths (Å)		0.01
RMSD bond angles(°)		1.22
***Average B-factors (Å^2^)***		
Protein atoms (2450 atoms)		67.4
Water molecules (51 atoms)		66.2
***Ramachandran plot***		
Most favored regions (%)		86.1
Additional allowed regions (%)		13.9
Generously allowed regions (%)		0
Disallowed regions (%)		0

Values in parentheses are for the last resolution shell (2.46−2.40 Å). The diffraction data were processed under the space group P1 and transformed to the space group C2 using the transformation A = 2c+a, B = a and C = b, where a,b,c and A,B,C are the P1 and C2 unit-cell vectors, respectively.

### Thrombin inhibition

All peptides were assayed for their abilities to inhibit thrombin amidolytic activity on chromogenic substrate S2238 (Chromogenix, Milano, Italy) as described previously [16], [17]. Values for concentration of peptide needed for 50% inhibition (*IC_50_*) and inhibition constant (*K_i_*) were calculated from data obtained were fitted using Origin software (MicroCal, Northampton, MA, USA). A detailed account for the selection and use of equations to fit the data is available in Materials and Methods S1.

### Zebrafish larvae venous thrombosis model

Zebrafish and the larvae were maintained as previously described [30]. Intravenous microinjections of peptides were performed using Nanoject II (Drummond, Broomall, PA, USA) with glass injection needles. Ten nanolitres of peptides or phosphate buffered saline (PBS) were injected into 4 days post-fertilization larvae through the posterior cardinal vein. Laser ablation of larval veins were performed with a pulsed nitrogen laser light pumped through coumarin 440 dye (445 nm) (MicroPoint Laser system, Photonic Instrument, St Charles, IL, USA) at 10 pulses/s with laser intensity setting at 10. Laser ablation of each larvae was carried out 20 min after microinjection of the peptide or PBS. The laser beam was aimed at the caudal vein around five somites towards the caudal end from the anal pore and triggered for 3 s. Thrombus formation following vein injury, due to laser ablation, was monitored and the time taken for complete occlusion of the injured vein was recorded.

## Results

### Thrombin:s-variegin structure

The crystal structure of the thrombin:s-variegin complex was determined at 2.4 Å resolution (Table 1 and Figure 1C–D). The electron density of the complex structure is well defined except for termini residues of chain A [^T^(^1H^TFGSGE^1C^) and ^T^Arg15]. The structure of thrombin in the complex superimposes well with other thrombin structures.

Only 17 out of the 32 residues (^V^His12 to ^V^Leu28) of s-variegin have well-defined density (Figure 1C). The first seven N-terminal residues do not make direct contact with thrombin [16] and s-variegin is cleaved by thrombin between ^V^Lys10 and ^V^Met11 [16]. It is likely that the N-terminal fragment ^V^(^1^SDQGDVAEPK^10^) has dissociated from thrombin after cleavage before crystallization. In contrast, the C-terminal fragment MH22 ^V^(^11^MHKTAPPFDFEAIPEEYLDDES^32^) remains bound to thrombin after cleavage [17]. The N-terminal ^V^Met11 and the last five residues ^V^(^28^LDDES^32^) of the fragment are not observed, reflecting disorder in the termini.

The C-termini of hirulog-1/-3, hirugen and hirudin have the following sequence DFEEIPEEYL(Q), with the Gln only present in hirudin. s-Variegin has an almost identical sequence ^V^(^19^DFEAIPEEYLDDES ^32^), with four extra residues in the C-terminus. Despite the identity, there are large differences between their conformations. The C-terminus is disordered in the hirulog-1/bivalirudin structure (PDB code: 2HGT) [4], forming a 3_10_ helix in hirulog-3 (PDB code: 1ABI) [22] and hirugen (PDB code: 1HGT [4]) and forming a full α-helical turn in sulfo-hirudin (PDB code: 2PW8) [31]. In s-variegin, these residues remain in an extended conformation until the last observed residues (^V^Leu28). The extra residues in C-terminus, although not observed in the present structure may cause the peptide to adopt the fully extended conformation (Figure S1).

### Interactions with thrombin catalytic residues

**Figure 2:**
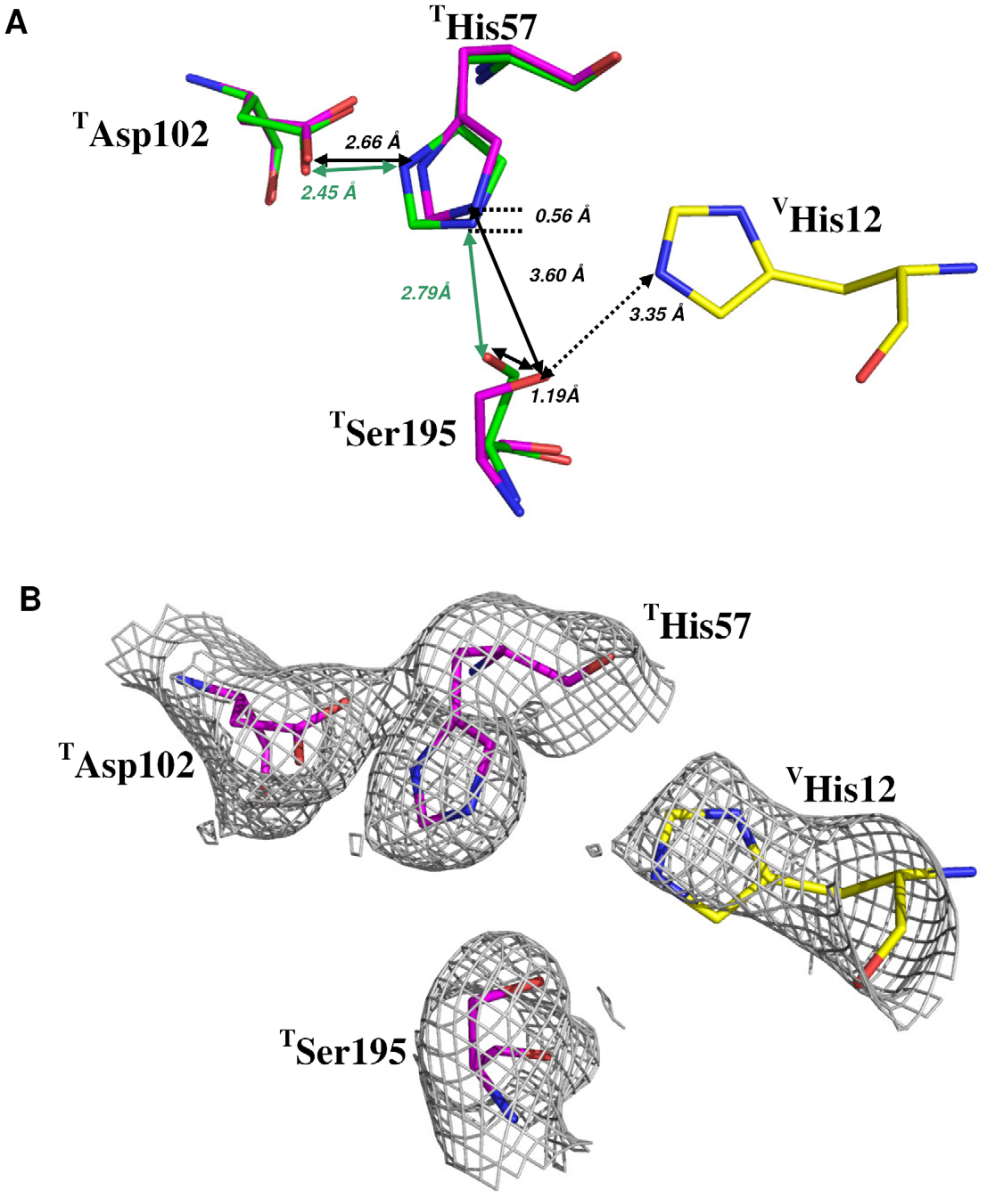
Thrombin catalytic triads in s-variegin-bound and hirugen-bound structures. (**A**) Thrombin catalytic triad ^T^His57, ^T^Asp102 and ^T^Ser195 in thrombin:hirugen structure (green) and in thrombin:s-variegin structure (pink) are superimposed. The ^T^Ser195 Oγ in thrombin:s-variegin structure is displaced by 1.19 Å compared to thrombin:hirugen structure. The displacement of ^T^Ser195 Oγ in thrombin:s-variegin structure (pink) is due to interactions with ^V^His12 of s-variegin through hydrogen bond (dotted arrow), rendering ^T^Ser195 a weak nucleophile that is incapable of catalysis. The imidazole ring of ^T^His57 also rotated, resulted in a displacement of its Nε by 0.56 Å. Overall, the distance between Nε of ^T^His57 and Oγ of ^T^Ser195 increases to 3.60 Å (black arrow) from 2.79 Å (green arrow), disrupts the catalytic charge relay system. (**B**) The 2Fo-Fc electron density map of thrombin catalytic triad and ^V^His12 contoured at 1.0σ.

The active site of thrombin in the crystal structure was compared to the published data for the thrombin:hirugen structure (unoccupied active site) (Figure 2). Of the three catalytic residues, the most striking differences are with the Oγ atom of ^T^Ser195 and the orientation of the imidazole ring of ^T^His57. In the thrombin:s-variegin structure, ^T^Ser195 Oγ is displaced by 1.19 Å, pointing towards s-variegin. Distance between ^T^Ser195 Oγ and the side chain Nε of ^V^His12 is 3.35 Å, possibly forming hydrogen bond (Table S1). At the same time, the distance between Nε of ^T^His57 and Oγ of ^T^Ser195 increases to 3.60 Å from 2.79 Å, breaking the crucial strong hydrogen bond needed to form the catalytic charge relay system. Without stabilization by the strong hydrogen bond between ^T^His57 and ^T^Ser195, the imidazole ring of ^T^His57 is now rotated slightly and leads to a displacement of its Nε by 0.56 Å (Figure 2A). The newly formed hydrogen bond between ^T^Ser195 and ^V^His12 delocalize the electrons of ^T^Ser195 Oγ, making ^T^Ser195 a weak nucleophile and incapable of efficiently attacking the backbone C of the substrate. This explains the observed classical non-competitive inhibition for MH22 [17].

### Interactions with prime subsites

**Figure 3:**
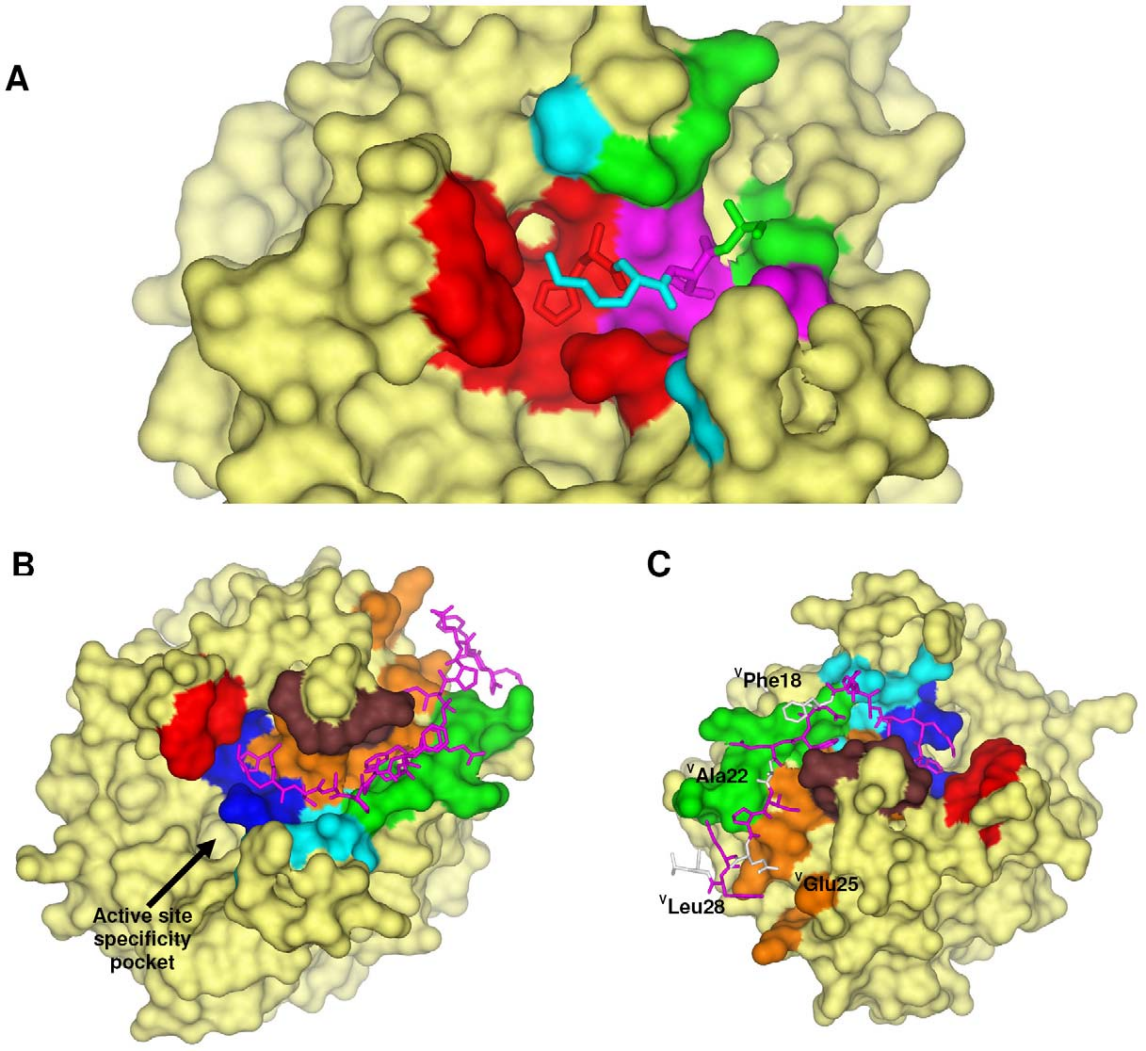
Interactions between thrombin and s-variegin. (**A**) Prime subsites interactions between thrombin and s-variegin (residues P2′ to P5′) are shown. Density for s-variegin P1′ ^V^Met11 cannot be traced in the structure. Thrombin S2′ subsite is colored in red, S3′ subsite in cyan, S4′ subsite in pink and S5′ subsite in green. (**B**) Thrombin residues that interfaced with s-variegin are colored according to their positions: catalytic pocket (blue): ^T^His57, ^T^Cys58, ^T^Cys191, ^T^Glu192, ^T^Gly193, ^T^Ser195; 60-loop (red): ^T^Trp60D and ^T^Lys60F; autolysis loop (cyan): ^T^Trp141, ^T^Gly142, ^T^Asn143, ^T^Thr147 and ^T^Gln151; 34-loop (brown): ^T^Phe34, ^T^Arg35, ^T^Gln38 and ^T^Glu39; 70-loop (green): ^T^Arg73, ^T^Thr74, ^T^Arg75, ^T^Tyr76 and ^T^Arg77A; bottom of the cleft (orange): ^T^Met32, ^T^Leu40, ^T^Leu41, ^T^Cys42, ^T^Leu65, ^T^Arg67, ^T^Lys81, ^T^Ile82, ^T^Met84 and ^T^Lys110. Sticks model of s-variegin is shown in pink. (**C**) All but four residues (^V^Phe18, ^V^Ala22, ^V^Glu25 and ^V^Leu28, white) on s-variegin have their side chains buried in the interface with thrombin.

In addition to the new hydrogen bond, the following interactions anchor s-variegin P2′ to P5′ residues ^V^(^12^HKTA^15^) to the thrombin prime subsites (Figure 3A). Besides the catalytic residues, ^T^Leu41, ^T^Cys42, ^T^Cys58, ^T^Trp60D, ^T^Lys60F and ^T^Glu192 are also in contact with ^V^His12. Two hydrogen bonds can be formed between ^V^His12 with ^T^Glu39 and ^T^Glu192 (Table S1). The P3′ (^V^Lys13) interacts with ^T^Arg35, ^T^Glu39, ^T^Trp60D, ^T^Lys60F, ^T^Asn143, ^T^Thr147, and ^T^Glu192. The P4′ (^V^Thr14) side chain is directed towards the base of the highly flexible autolysis-loop. The side chain occupies a surface lined by ^T^Leu40, ^T^Trp141, ^T^Gly142, ^T^Asn143, ^T^Gln151 and ^T^Gly193. Interactions within this P3′ subsite are strengthened by two hydrogen bonds between ^V^Thr14 with ^T^Asn143 and ^T^Gln151 (Table S1). The P5′ ^V^Ala is surrounded by ^T^Gln38, ^T^Glu39, ^T^Arg73 and ^T^Gln151. Thus there are extensive interactions between the variegin peptide and thrombin prime subsites.

### Interactions with exosite-I

s-Variegin fits firmly into the canyon-like cleft extending from the thrombin active site to exosite-I. The walls of this hydrophobic cleft are formed by the 60- and autolysis- loops near the active site, and 34- and 70- loops at exosite-I, while many apolar residues in these loops line the bottom [2], [3]. s-Variegin is in close contact with multiple residues in exosite-I as depicted in Figure 3B. All but four residues of s-variegin (^V^Phe18, ^V^Asp19, ^V^Ala22 and ^V^Glu26) have their side chains buried in the interfaces with thrombin (Figure 3A).

**Figure 4:**
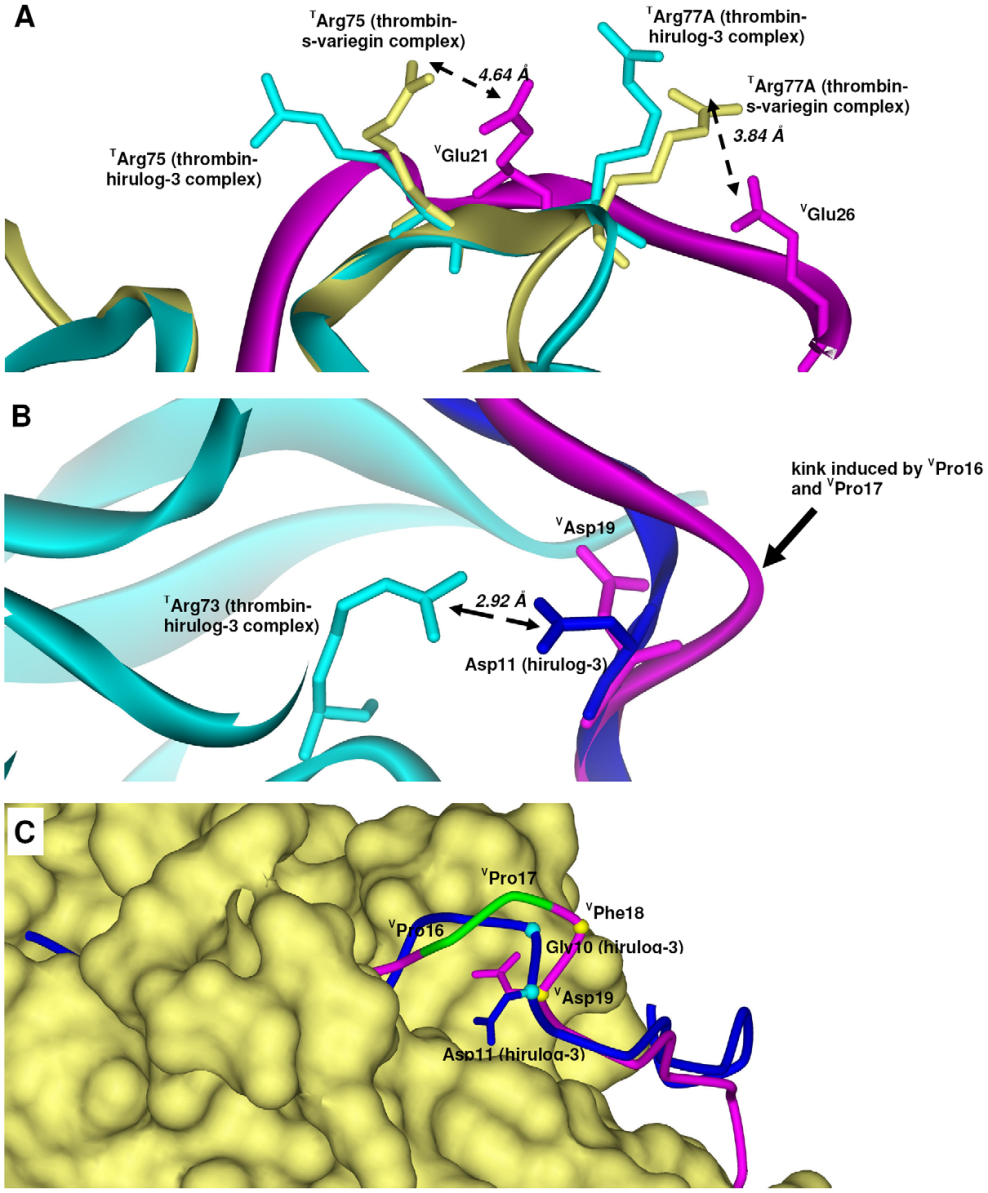
Electrostatic interactions in thrombin:s-variegin structure. (**A**) s-Variegin and hirulog-3 have distinct ion pairs formed with exosite-I of thrombin despite high sequence identity. A salt bridge (3.84 Å) between ^V^Glu26 (pink) and ^T^Arg77A (yellow) is absent in hirulog-3 as ^T^Arg77A (cyan) points away from the inhibitor. Weak salt bridge (4.64 Å) is also likely between ^V^Glu21 (pink) and ^T^Arg75 (yellow) rotated 90.5° about Cβ compared to ^T^Arg75 in hirulog-3 bound thrombin (cyan) to facilitate interaction with ^V^Glu21 (pink). Electron density maps of residues involved are shown in Figure S2A. (**B**) The strong ion pair (Asp11∶^T^Arg73, 2.92 Å) in thrombin:hirulog-3 structure is absent in thrombin:s-variegin structure since ^V^Asp19 (pink) pointed to an opposite direction compared to the analogous hirulog-3 Asp11 (blue) due to a kink in s-variegin backbone (pink). Electron density maps of residues involved are shown in Figure S2B. (**C**) The presence of a ^V^Pro16-^V^Pro17 (green) in s-variegin resulted in the kink. Superimposition of s-variegin (pink, only Cα positions traced) and hirulog-3 (blue, only Cα positions traced) based on their thrombin structures showed displacement of ^V^Phe18 and ^V^Asp19 from their corresponding residues Gly10 and Asp11 of hirulog-3 by 3.11 Å and 0.79 Å (measured by Cα positions), respectively. As a result, the distance between ^T^Arg73 and ^V^Asp19 charges are 5.83 Å, rendering electrostatic interactions impossible.

Interestingly, the high identity between C-terminus of s-variegin, hirulog-1/-3, hirugen and hirudin are not reflected in their respective salt bridges formation with exosite-I of thrombin. Despite the presence of multiple anionic residues in the s-variegin C-terminus and highly cationic exosite-I, only one strong salt bridge is formed (^V^Glu26∶^T^Arg77A). This salt bridge (3.84 Å) is not observed in hirulog-1/-3, hirugen and hirudin structures as ^T^Arg77A adopts a different rotamer that points away from the inhibitor (Figure 4A and S2A). In addition, a weak salt bridge is also likely between ^V^Glu21 and ^T^Arg75 (4.64 Å). In hirulog-1/-3, hirugen and hirudin structures the analogous Glu makes an ion pair with ^T^Arg75 of a 2 fold symmetry-related thrombin, although this interaction is suggested to occur within the same thrombin:inhibitor pair in solution [4], [22], [32]. In our structure, the ^T^Arg75 side chain is rotated by 80.5° about Cβ, compared to the thrombin:hirulog-3 complex (Figure 4A and S2A) facilitating this interation. In hirulog-1/-3 and hirugen structures, an ion pair between ^T^Arg73 and the Asp, analogous to ^V^Asp19, can be observed. However, formation of this ion pair in the thrombin:s-variegin complex is not possible as the ^V^Asp19 side chain points in an opposite direction into solvent. This difference is most likely due to the kink in the s-variegin backbone, induced by ^V^Pro16-^V^Pro17 (see below) (Figure 4B–C and S2B).

The end of the canyon-like cleft is a relatively flatter surface and formed by ^T^Asp63 to ^T^Ile68 and ^T^Lys81 to ^T^Leu85. The residues ^V^Pro24 to ^V^Tyr27 are stacked loosely on top of this surface with one of the side chains (^V^Glu25) pointing towards solvent (Figure 3C). This s-variegin segment is in a different conformation when compared to hirulog-3/hirugen despite sequence similarity (Figure S1).

### Design and characterization of variegin variants

Several new variegin variants were designed based on the thrombin:s-variegin structure, as well as background information available on thrombin:inhibitor interactions.

- **Optimization of the length of variegin.** The lack of electron density for the last four C-terminal residues [^V^(^29^DDES^32^)] of s-variegin in the complex structure indicates flexibility and hence lack of close contacts with thrombin. As these residues are also not present in hirulogs or hirugen, two variants (EP21 and MH18) lacking the last four C-terminal residues were synthesized and characterized. *IC_50_* and *K_i_* values of EP21 and MH18 are essentially identical with their templates (EP25 and MH22, respectively) and indicate that the truncation of the four C-terminal residues does not alter the inhibitory activity (Table 2).

**Table 2:** Sequence and activity of variegin and its variants.

Name	Sequence	Pre-incubation tine (min)	*IC_50_* (nM)	*K_i_* (nM)	Mechanism	Plots shown in figure
s-variegin	SDQGDVAEPKMHKTAPPFDFEAIPEEYLDDES	0	8.25±0.45	0.318±0.020	Fast, tight-binding, competitive	Published [17]
		20	10.4±0.3			
EP25	SDQGDVAEPKMHKTAPPFDFEAIPEEYLDDES	0	173±26	0.365±0.109	Slow, tight-binding, competitive	S3
		20	13.1±0.7			
MH22	SDQGDVAEPKMHKTAPPFDFEAIPEEYLDDES	0	11.5±0.7	14.1±0.3	Fast, tight-binding, noncompetitive	Published [17]
		20	12.3±1.9			
Hirulog-1	_D_ FPRPGGGGNGDFEEIPEEYL	0	72.6±3.9	2.94±0.12	Fast, tight-binding, competitive	Published [17]
		10	102±13			
EP21	SDQGDVAEPKMHKTAPPFDFEAIPEEYLDDES	0	177±7	0.315±0.024	Slow, tight-binding, competitive	S4
		20	16.2±2.9			
MH18	SDQGDVAEPKMHKTAPPFDFEAIPEEYLDDES	0	10.9±1.2	14.9±3.5	Fast, tight-binding, noncompetitive	S5
		20	11.7±1.9			
DV24	SDQGDVAEPKMHKTAPPFDFEAIPEEYLDDES	0	7.49±0.28	0.306±0.029	Fast, tight-binding, competitive	S7
		20	10.1±0.6			
DV24*H12A*	SDQGDVAEPKMAKTAPPFDFEAIPEEYLDDES	0	48.2±12.4	3.23±0.48	Fast, tight-binding, competitive	S8
		20	141±11			
MH18*H12A*	SDQGDVAEPKMAKTAPPFDFEAIPEEYLDDES	0	328±23	329±8	Fast, tight-binding, noncompetitive	S9
		20	343±46			
DV24*K10R*	SDQGDVAEPRMHKTAPPFDFEAIPEEYLDDES	0	6.98±0.76	0.259±0.015	Fast, tight-binding, competitive	S10
		20	12.0±0.4			
DV23	SDQGDVAEPKMHKTAPPFDFEAIPEEYLDDES	0	45.4±1.6	2.19±0.23	Fast, tight-binding, competitive	S11
		20	77.8±6.1			
DV23*K10R*	SDQGDVAEPRMHKTAPPFDFEAIPEEYLDDES	0	12.9±1.0	0.600±0.010	Fast, tight-binding, competitive	S12
		20	102±1			
EP25*A22E*	SDQGDVAEPKMHKTAPPFDFEEIPEEYLDDES	0	124±23	0.311±0.070	Slow, tight-binding, competitive	S13
		20	13.5±2.1			
MH22*A22E*	SDQGDVAEPKMHKTAPPFDFEEIPEEYLDDES	0	13.6±0.5	15.1±1.0	Fast, tight-binding, noncompetitive	S14
		20	15.6±0.4			
DV24*Y^phos^*	SDQGDVAEPKMHKTAPPFDFEAIPEEY^¶^LDDE	0	8.67±0.45	0.327±0.032	Fast, tight-binding, competitive	S15
		20	12.4±1.2			
DV24*K10RY^phos^*	SDQGDVAEPRMHKTAPPFDFEAIPEEY^¶^LDDES	0	4.64±0.78	0.150±0.018	Fast, tight-binding, competitive	S16
		20	7.80±1.80			
DV24*Y^sulf^*	SDQGDVAEPKMHKTAPPFDFEAIPEEY*LDDE	0	1.66±0.18	0.0560±0.0180	Fast, tight-binding, competitive	S17
		20	2.02±0.29			
DV24*K10RY^sulf^*	SDQGDVAEPRMHKTAPPFDFEAIPEEY*LDDE	0	1.39±0.17	0.0420±0.0061	Fast, tight-binding, competitive	S18
		20	1.66±0.21			
MH18*Y^sulf^*	SDQGDVAEPKMHKTAPPFDFEAIPEEY*LDDES	0	1.26±0.18	1.25±0.18	Fast, tight-binding, noncompetitive	S19
		20	1.17±0.14			

Y^¶^: phosphotyrosine; Y*: sulfotyrosine.

Previously, we have shown that the first seven N-terminal residues of variegin [^V^(^1^SDQGDVA^7^)] are responsible for its fast-binding kinetics, due to a possible steering effect towards the highly basic thrombin exosite-II [16]. EP25 and EP21, in which these residues are removed, act as slow-binding inhibitor. For complete inhibition, they required 20 min of pre-incubation with thrombin. Since exosite-II is located about 10 Å away from the active site [33], we extended EP21 by three residues at the N-terminal to include one of the two acidic residues, ^V^Asp5, in DV24. The non-linear progress curves of thrombin inhibition by EP21 (characteristic of slow binding inhibitors), changed to linear progress curves of inhibition by DV24 (characteristic of fast binding inhibitor) (Figure S6). The *IC_50_* and *K_i_* values of DV24 are identical to those of s-variegin (Table 2). Like for s-variegin, a fast binding inhibitor, the *IC_50_* of DV24 increases with pre-incubation due to the cleavage by thrombin. Thus, DV24 is eight residues shorter than s-variegin but retains fast-binding kinetics and potency.

- **Optimization of thrombin:variegin interactions.** As observed in the thrombin:s-variegin structure, ^V^His12 binds to the prime subsite, with its side chain nitrogen forming hydrogen bond with ^T^Ser195 and disrupting the charge relay system of the thrombin catalytic triad. In order to verify the significance of ^V^His12, two variants were synthesized by replacing this residue with Ala. The two variants, DV24*H12A* and MH18*H12A* are based on the sequences of DV24 and MH18, repectively and represent the minimal interacting sequences of variegin and cleaved fragment. Both peptides lose their inhibitory potency significantly. *IC_50_* (∼6 fold) and *K_i_* (∼10 fold) values of DV24*H12A* increase when compared to DV24. Pre-incubation of DV24*H12A* with thrombin also causes a larger increase in *IC_50_* (∼3 fold in 20 min) when compared to DV24 (<2 fold) (Table 2).

MH18*H12A* shows a more drastic increase in *IC_50_* (∼30 fold) and *K_i_* (∼22 fold) values, compared to MH18 (Table 2). Maximum inhibition by MH18*H12A* appears to saturate near 80% (highest concentration used is 30 µM), implying that the peptide is unable to completely inhibit thrombin. Thus, the single mutation of ^V^His12 to Ala significantly affects the inhibitory action of the peptide, ascertaining the importance of ^V^His12, as observed in the structure of thrombin:s-variegin complex. However, the activity is not completely abolished possibly due to other interactions in the prime subsites retained in these alanine mutants.

One striking difference between variegin and other thrombin substrates/inhibitors is the presence of Lys, instead of Arg, at P1. Typically, P1 Lys interacts with ^T^Asp189 through a water molecule, resulting in reduced affinity and specificity [34], [35]. Therefore, using DV24 as a template sequence, the P1 residue (^V^Lys10) was replaced by Arg in DV24*K10R*. The peptide has marginally improved *IC_50_* and *K_i_* values, when compared to DV24 (Table 2). Substitution of P1 Lys by Arg appears to accelerate the cleavage, as shown by the higher *IC_50_* after 20 min pre-incubation, when compared to DV24 (Table 2).

As in hirulogs, hirugen and hirudin, the phenyl group of ^V^Phe20 interacts with ^T^Phe34 through π-stacking. In s-variegin, there are nine residues between this ^V^Phe20 and the P1 residue, ^V^(^11^MHKTAPPFD^19^), unlike in hirulog-1/-3, which has only eight residues (^4^PGGGGNGD^11^). ^V^Pro16 and ^V^Pro17 induce a kink in the s-variegin backbone, causing a slight bend upwards, away from thrombin (Figure 4B). This, in turn, causes displacement of ^V^Phe18 and ^V^Asp19 by about 3.11 Å and 0.79 Å (based on Cα positions), respectively against their analogs in hirulog-3 (Gly10 and Asp11). Crucially, Asp11 of hirulog-3 makes an ion pair with ^T^Arg73, while analogous ^V^Asp19 does not (Figure 4B). In fact, the ^V^Asp19 side chain points in the opposite direction (towards solvent), with a 5.83 Å distance between ^V^Asp19 and ^T^Arg73 (Figure 4C). To remove the kink in the backbone, reposition ^V^Asp19 and create the ionic interaction, ^V^Pro16 was deleted in variants DV23 and DV23*K10R*. However, DV23 shows an average of ∼7 fold reduction in *IC_50_* when compared to DV24 (Table 2). The other variant, DV23*K10R*, is also less active when compared to DV24*K10R*, albeit to a lesser extent (Table 2). *IC_50_* values of both DV23 and DV23*K10R* significantly increased upon pre-incubation, implies that the cleaved products no longer potently inhibits thrombin (Table 2). In addition, the peptide with Arg at P1 (DV23*K10R*) is hydrolyzed by thrombin at a faster rate than the peptide with Lys at P1 (DV23) judging from the more rapid increase of *IC_50_* values with pre-incubation (Table 2). Thus, the deletion of ^V^Pro16 has an adverse effect on the activities of both the intact and cleaved peptides. This deletion probably compromises the interactions of P′ residues with the prime subsites, owing to their proximity to ^V^Pro16.

In hirudin, ^H^Glu58 makes an ion-pair with ^T^Arg77A [3], [32]. However, in variegin, this Glu is replaced by ^V^Ala22 and its side chain is solvent exposed (Figure 3C). ^V^Ala22 was replaced by Glu in variants EP25*A22E* and MH22*A22E*. The four C-terminal residues were retained in these variants to maintain the original micro-environment near the C-terminus. *IC_50_* and *K_i_* values of EP25*A22E* and MH22*A22E* are similar to their templates (EP25 and MH22, respectively) (Table 2). Thus, the replacement of ^V^Ala22 by Glu does not enhance the activity of variegin.

Desulfation of Tyr63 in hirudin or hirugen is known to reduce their affinities to thrombin by about 10 fold [36]–[38]. Interestingly, the analogous residue in native variegin, ^V^Tyr27, is not sulfated. We postulated that modification of ^V^Tyr27 could also increase its binding affinity towards thrombin. Considering the similarity of phosphate and sulfate moieties (similar size and overall negative charge), phosphotyrosine and sulfotyrosine residues were incorporated to design new variants.

One phosphotyrosine residue was added to DV24 and DV24*K10R* to produce the variants DV24*Y^phos^* and DV24*K10RY^phos^*, respectively. DV24*Y^phos^* is marginally less active than DV24, whereas DV24*K10RY^phos^* has slightly improved activity (Table 2).

Similarly, a sulfotyrosine residue was incorporated in three new variants, DV24Y*^sulf^*, DV24*K10R*Y*^sulf^* and MH18Y*^sulf^*. DV24Y*^sulf^* and DV24*K10R*Y*^sulf^* show an average ∼5 fold increase in *IC_50_* and *K_i_* values when compared to the respective non-sulfated variants, DV24 and DV24*K10R*, respectively (Table 2). MH18Y*^sulf^* also has improved activity when compared to MH18 (Table 2). It is very likely that the presence of sulfo-Tyr27 and the truncation of extra residues in variegin variants cause a rearrangement of the C-terminal conformation to mimic the hirugen/hirudin C-termini. Strong affinities are obtained in these variants through optimization of C-terminal interactions.

**Figure 5:**
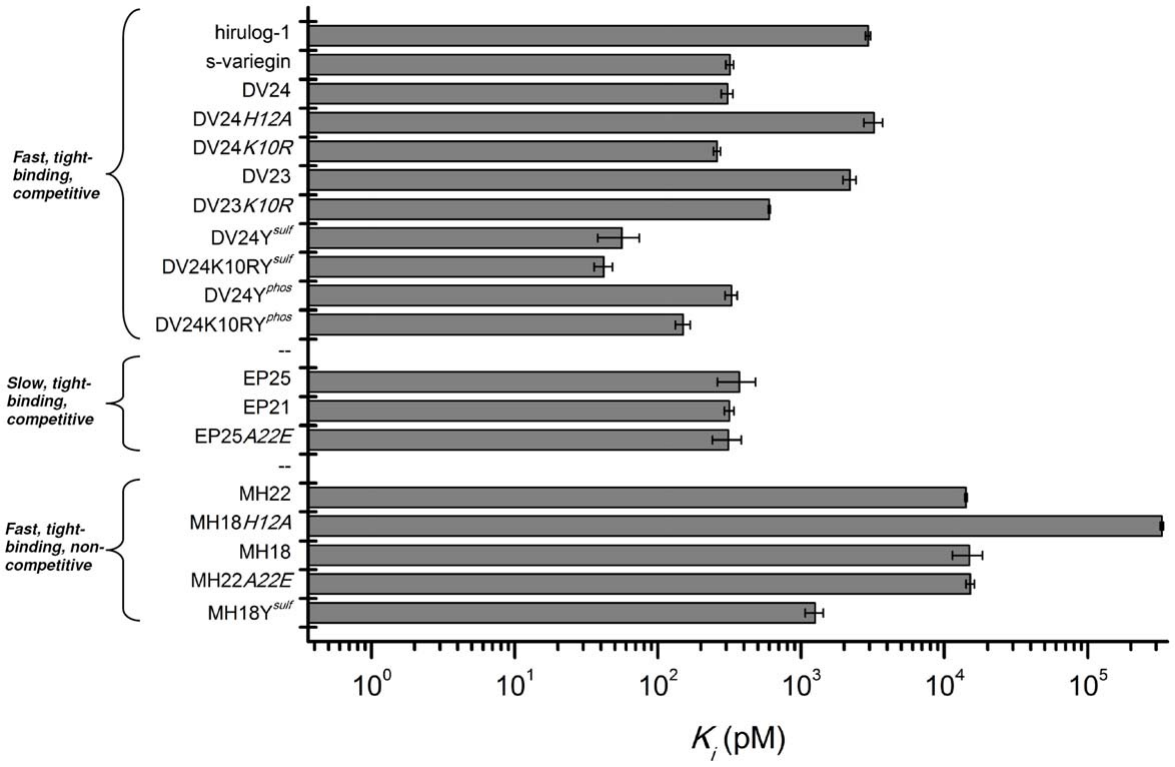
*K_i_* values of all peptides (including hirulog-1/bivalirudin). Peptides are grouped according to their mechanism of actions. All competitive inhibitors (fast or slow) have higher affinities to thrombin compared to hirulog-1/bivalirudin. The most potent variant DV24*K10RY^sulf^* is about 70-fold stronger. Even their cleavage products (non-competitive inhibitors) are potent inhibitor, with one of them, MH18*Y^sulf^*, binds to thrombin approximately 2-fold tighter than hirulog-1/bivalirudin.

Figure 5 shows the plot of *Ki* values of all peptides (including hirulog-1/bivalirudin). Affinity of DV24*K10RY^sulf^* for thrombin (*K_i_* = 42.0±6.1 pM) is ∼70 fold stronger than that of hirulog-1/bivalirudin (*K_i_* = 2.94±0.12 nM). Based on the derived structure-activity relationships, we have designed a shorter (24-mer DV24*K10RY^sulf^* against 32-mer s-variegin) yet more potent thrombin inhibitor (*K_i_* of 42 pM compared to 318 pM).

### *In vivo* antithrombotic effects of the peptides

Five inhibitors were selected as representatives to test for their antithrombotic effects *in vivo* using the zebrafish larvae venous thrombosis model: (1) s-variegin, the full-length variegin, a fast and tight-binding competitive inhibitor; (2) EP25, without seven N-terminal residues, has similar affinity for thrombin, but is a slow and tight-binding competitive inhibitor; (3) MH22, the cleaved product that is a fast and tight-binding, noncompetitive inhibitor; (4) DV24*K10RY^sulf^*, the most potent *in vitro* inhibitor that is a fast and tight-binding competitive inhibitor and (5) hirulog-1/bivalirudin, a fast, tight-binding, competitive inhibitor currently in clinical use. Hirulog-1/bivalirudin was used as a positive control.

**Figure 6:**
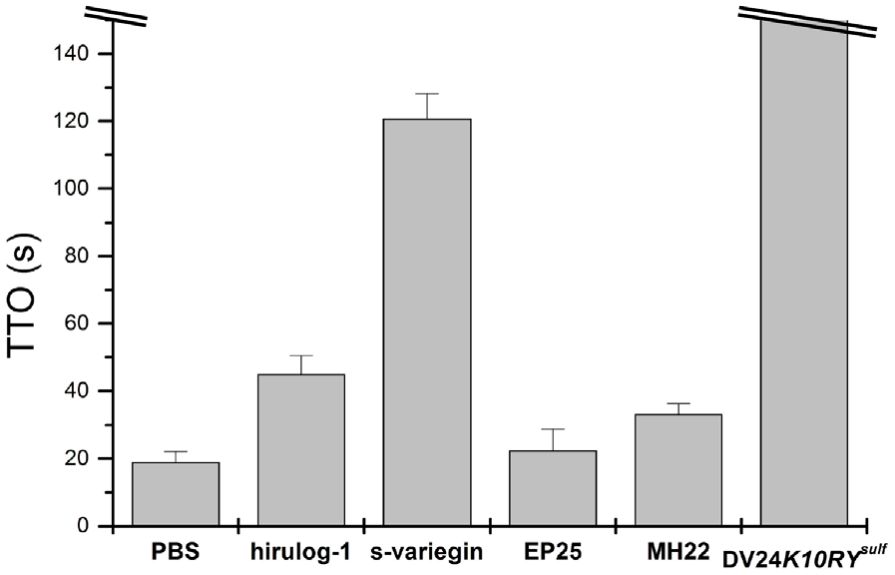
*In vivo* antithrombotic effects of peptides. Zebrafish 4 days post-fertilization larvae were injected with 10 nl of different peptides at 500 µM or 10 nl of PBS as control. TTO for larvae injected with PBS, hirulog-1/bivalirudin, s-variegin, EP25 and MH22 are 19.0±3.2 s, 45.0±5.5 s, 120.8±7.4 s, 22.5±6.2 s and 33.3±2.9 s, respectively. Within 150 s, no thrombus was formed in larvae injected with DV24*K10RY^sulf^*. With the exception of the slow binding inhibitor EP25, the abilities of the peptides to prolong TTO correlate with their *in vitro K_i_* (n = 4, error bars represent S.D.).

All five peptides were injected into the zebrafish larvae circulation through the posterior (caudal) cardinal vein at a single dose (500 µM, 10 nl). The antithrombotic effects of the peptides were measured as the delay in time-to-occlusion (TTO) of the caudal vein after laser ablation (Figure 6). Overall, other than EP25, the antithrombotic effects of the peptides correlated well with their affinities for thrombin. Thus, the slow-binding inhibitory mode (EP25) is not desirable for *in vivo* efficacy while both fast, competitive (s-variegin, DV24*K10R*Y*^sulf^* and hirulog-1/bivalirudin) and fast, noncompetitive (MH22) inhibitors are effective. Our results are consistent with similar observations reported earlier about the importance of rapid thrombin inhibition for efficacious antithrombotic agents [39].

## Discussion

Variegin belongs to a unique class of thrombin inhibitors that have potential as antithrombotic agents [16], [17]. We solved the structure of thrombin:s-variegin complex at 2.4 Å resolution. Despite the use of full-length s-variegin for co-crystallization, only the density of its C-terminal fragment was observed. The cleaved fragment (equivalent to MH22) stays bound tightly to the prime subsites and exosite-I, exhibiting prolonged inhibitory action (>18 h) [17].

Active site inhibitors of thrombin typically target the non-prime subsites, hindering the access of substrates (including the chromogenic substrate S2238 used in this study) to the catalytic residues [29], [31], [40]. The full-length variegin also acts in a similar way and competitively inhibits the thrombin active site [16]. The present thrombin:s-variegin structure reveals two exciting features: (1) the novel mechanism of thrombin inhibition through disruption of the charge relay system, and (2) the binding with thrombin prime subsites. The cleaved product (MH22) still retains inhibition through this novel mechanism, as observed in the structure. The lack of overlaps in MH22 and S2238 binding sites (non-prime and S1′ subsites), demonstrates the feasibility of simultaneous binding of S2238 and MH22 to thrombin with no changes in affinities of either (Figure S20). The binding of substrate (S2238) to the thrombin active site becomes non-productive due to the disruption of the charge relay system by MH22 (Figure 2). We also speculate that the binding of variegin/MH22 to the prime subsites induces minor structural changes that may interfere with the entry of S2238 or exit of products from the active site without affecting the strength of substrate binding. Thus the structure explains the classical noncompetitive inhibition by MH22.

The interaction between the variegin and thrombin prime subsites is equally interesting. The S′ subsites' interactions between the inhibitors and protease are important for binding [41]. A systematic probing of thrombin S′ subsites with ‘methyl scan’ also has demonstrated the potential for targeting prime subsites in the design of inhibitors [42]. However, unlike variegin, other naturally occurring thrombin inhibitors, including hirudin [31], rhodniin [43], ornithodorin [44] and boophilin [45], are not inserted into the canyon-like cleft (the prime subsites) connecting the active site and exosite-I. Similarly, the previous structures of thrombin, in complex with other macromolecular substrates [46]–[50], typically lack complete details in the interactions within this region. Thus, when compared to the non-prime subsites, understanding of the binding preferences for the thrombin prime subsites (especially S2′ and beyond) is much less complete [1]. Recently, the structure of a PAR1 fragment in complex with an inactive thrombin mutant ^T^Ser195A was published [51]. This structure provided new details on the interaction within the prime subsites. However, there is considerable disorderliness in the PAR1 molecule binding to this region. As a result, it was suggested that the prime subsites binding segment of PAR1 snaps away from thrombin upon cleavage [51]. In the case of substrates, such a mechanism is probably advantageous to ensure a faster turnover. However, inhibitors are developed to bind to thrombin as tight and long as possible. In this specific case, variegin has evolved to bind to the thrombin prime subsites and thus the structure of the thrombin:s-variegin complex provides a unique opportunity to understand the interaction of this inhibitor with the thrombin prime subsites which is of extreme importance to the design of more effective inhibitors.

In hirulog-1/bivalirudin, the glycyl linkers connect the active site and exosite-I binding moieties without displaying specific interactions with the thrombin prime subsites. As a result, this segment is disordered in the crystal structure [4] and the activity is rapidly lost after cleavage by thrombin [17], [52]. When anchored by a non-hydrolyzable active site binding moiety and an exosite-I binding segment, the non-specific linker is forced to fit into the canyon-like cleft in the prime subsites, as seen in hirulog-3 [22] and P498 [40]. In these cases, the S′ subsite interactions are sub-optimal due to the lack of specific side chain interactions. As a result, extensive and lengthy optimizations, through synthetic chemistry using multiple and unnatural amino acids, were necessary to produce inhibitors with enhanced binding to the prime subsites [42], [53], [54]. In contrast, tight binding of s-variegin to the prime subsites is achieved through specific interactions involving the side chains of natural amino acids. Thus the thrombin-variegin complex provides important and detailed structural information for inhibition of the thrombin prime subsites. This structural observation for the prime subsites binding in variegin is also consistent with the structure-function data presented here and elsewhere [16], [17]. The firm insertion of variegin peptides into the cleft in an extended conformation is probably the simplest structure needed to achieve strongest and instantaneous binding to the thrombin catalytic pocket, prime subsites and exosite-I. This ‘minimalistic’ approach in nature (ticks) confers an advantage of minimum energy expenditure (protein synthesis) for maximum outcome (potent inhibition of coagulation enzymes to facilitate blood-feeding).

Based on the thrombin:s-variegin structure and other available information, we performed targeted structure-function relationship studies on variegin. Substitution of P1 Lys by Arg is reported to increase affinity for thrombin by ∼10 fold through a better fit to the S1 subsite [2], [35]. However, our results show that the improvement in affinity by this substitution varies. In DV24, DV24*Y^sulf^* and DV24*Y^phos^*, the effects of substitution are less than 2 fold. It is likely that the extensive interactions between the s-variegin P′ residues and thrombin S′ subsite compensate for the loose fitting of P1 Lys. In contrast, DV23/DV23*K10R*-thrombin interactions around the prime subsites are likely to be disturbed due to the deletion of Pro16 (DV23 *K_i_* is ∼7 fold higher than DV24). In this situation, P1 Arg facilitates stronger binding of the ^V^(^5^DVAEPR^10^) sequence to the active site compared to P1 Lys, which is reflected by the higher gain in affinity (∼4 fold decrease in *K_i_*). These results further support the importance of prime subsite interactions.

We have also shown that addition of three residues at the N-terminus of the slow-binding inhibitor EP21 changes it to a fast-binding inhibitor. Thus, the prime subsites anchoring effect (discussed above) mainly drives affinity for the thrombin active site, while the N-terminal steering effect is needed for proper pre-orientation of this segment. Hence, the P1 to P3 residues of s-variegin are inserted rapidly into the thrombin active site, assisted by prime subsites targeting and the N-terminal negative charge. Once in the acidic S1 pocket, P1 Lys/Arg interacts with ^T^Asp189 with only a minimal overall loss of binding strength. This suggests a less stringent requirement for the P1 residue that can be exploited for the design of new specific thrombin inhibitors. In addition to the prime subsite interaction, ^V^His12 draws ^T^Ser192 out of position and affects the geometry of the crucial catalytic residue as well as interfering in the charge relay system. The result is decreased catalytic efficiency. Such a design may be exploited in the development of inhibitors for other serine proteases.

The new variegin variants cover a big range of potency, speed of onset and kinetic parameters, showing the potential to ‘tune’ variegin to provide different therapeutic properties. The unique ability of variegin to potently inhibit thrombin (initially competitive, subsequently noncompetitive) for a long duration represents a new approach to anticoagulation when compared to other direct thrombin inhibitors on the market. Variegin and some of its variants inhibit thrombin potently with low *K_i_* (between 0.04 to 0.4 nM). Their affinities for thrombin are stronger than hirulog-1/bivalirudin (*K_i_* = 2.3 nM) [55], argatroban (*K_i_* = 3.2 nM) [56] and dabigatran (*K_i_* = 4.5 nM) [57] but weaker than hirudin (*K_i_* = 0.2 pM) [36]. While weaker affinities may translate to lower efficacy and increased probability of side effects, the almost irreversible binding of hirudin to thrombin (*K_i_* = 0.2 pM) may be responsible for an increased risk of major bleeding when compared to unfractionated heparin [58]. Thus, in terms of affinity for thrombin, variegin may represent a good balance between potency and safety. The prolonged action of variegin might also allow single dose administration instead of continuous infusion (as in the case of hirulog-1/bivalirudin) [11] especially in short procedures such as percutaneous coronary intervention. We also have preliminary data showing the possibility of using protamine as antidote for variegin. Overall, variegin (with its variants) represents a fine balance between hirudin and hirulog-1/bivalirudin for most of their properties (*K_i_*, size, duration of action etc.). At this point, the strong *in vivo* antithrombotic effects support the continual development of variegin and its variants as potential and improved anticoagulants.

## Supporting Information

Figure S1 **Conformation of s-variegin C-terminus.** s-Variegin C-terminus (pink) has a vastly different conformation compared to hirulog-1, hirulog-3, hirugen and sulfo-hirudin: (**A**) Residues PEEYL in hirulog-1 (red) are disordered and missing from the structure. (**B**) Residues PEEYL in hirulog-3 (blue) form a 3_10_ helix turn. (**C**) These residues in hirugen (green), with sulfated tyrosine, also form a 3_10_ helix turn. (**D**) Other than Tyr-sulfation, sulfo-hirudin (cyan) C-terminus has an extra Gln, forms a full α-helical turn.
(TIF)

Figure S2 **Electrostatic interactions in thrombin:s-variegin structure.** (**A**) Figure shows the electron density map (2Fo-Fc, 0.9σ) of residues described in Figure 4A in the main manuscript. Thrombin is colored yellow and s-variegin is colored pink. Map for thrombin colored in light cyan and map for s-variegin colored in gray. Residues involved in forming salt bridges are labeled. (**B**) Figure shows the electron density map (2Fo-Fc, 0.9σ) of residues described in Figure 4B in the main manuscript. Thrombin is colored yellow and s-variegin is colored pink. Map for thrombin colored in light cyan and map for s-variegin colored in gray.
(TIF)

Figure S3 **Variegin variant EP25 (slow binding, competitive inhibitor).** (**A**) Dose response curves of thrombin (1.65 nM) inhibited by EP25 (0.1 nM, 0.3 nM, 1 nM, 3 nM, 10 nM, 30 nM, 100 nM, 300 nM, 1000 nM, 3000 nM) in S2238 (100 µM) showed a left shift with increased pre-incubation time due to slow binding. *IC_50_* are 173±26 nM without pre-incubation (▪ solid line) and 13.1±0.7 nM with 20 min pre-incubation (○ dotted line) (n = 3, error bars represent S.D.). (**B**) Progress curves (not shown) of thrombin (0.8 nM) inhibited by EP25 (9.4 nM, 12.5 nM, 18.8 nM, 25 nM, 37.5 nM, 50 nM, 75 nM and 100 nM) in S2238 (100 µM) were fitted to equation **(6)** describing a slow binding inhibitor to obtain a *k* for each concentrations of EP25. Plot of *k* against EP25 concentrations (▪ solid line) is hyperbolic and fitted to equation **(7)** producing *K_i_′* of 0.882±0.128 nM, representing the dissociation constant of initial collision complex EI (**scheme 1**). *K_i_* calculated from equation **(8)** is 0.365±0.109 nM (n = 3, error bars represent S.D.).
(TIF)

Figure S4 **Variegin variant EP21 (slow binding, competitive inhibitor).** (**A**) Dose response curves of thrombin (1.65 nM) inhibited by EP21 (0.3 nM, 1 nM, 3 nM, 10 nM, 30 nM, 100 nM, 300 nM, 1000 nM, 3000 nM and 10000 nM) in S2238 (100 µM) showed a left shift with increased pre-incubation time due to slow binding. *IC_50_* are 177±7 nM without pre-incubation (▪ solid line) and 16.2±2.9 nM with 20 min pre-incubation (○ dotted line) (n = 3, error bars represent S.D.). (**B**) Progress curves (Figure S4) of thrombin (0.8 nM) inhibited by EP21 (18.8 nM, 25 nM, 37.5 nM, 50 nM, 75 nM, 100 nM and 150 nM) in S2238 (100 µM) were fitted to equation **(6)** describing a slow binding inhibitor to obtain a *k* for each concentrations of EP21. Plot of *k* against EP21 concentrations (▪ solid line) is hyperbolic and fitted to equation **(7)** producing *K_i_′* of 1.66±0.36 nM, representing the dissociation constant of initial collision complex EI (**scheme 1**). *K_i_* calculated from equation **(8)** is 0.315±0.024 nM (n = 3, error bars represent S.D.).
(TIF)

Figure S5 **Variegin variant MH18 (fast, tight-binding, noncompetitive inhibitor).** (**A**) Dose response curves of thrombin inhibition (1.65 nM) by MH18 (0.1 nM, 0.3 nM, 1 nM, 3 nM, 10 nM, 30 nM, 100 nM, 300 nM, 1000 nM, 3000 nM and 10000 nM) in S2238 (100 µM) are independent of pre-incubation time. *IC_50_* are 10.9±1.2 nM without pre-incubation (▪ solid line) and 11.7±1.9 nM with 20 min pre-incubation (○ dotted line) (n = 3, error bars represent S.D.). (**B**) Thrombin (1.65 nM) inhibition was tested with MH18 (0.39 nM, 0.78 nM, 1.56 nM, 3.13 nM, 6.25 nM, 12.5 nM, 25 nM, 50 nM, 100 nM and 200 nM) in S2238 (100 µM) (▪ solid line). Apparent inhibition constant *K_i_′* obtained by fitting data to equation **(2)**, describing fast and tight-binding inhibitor, is 14.9±3.5 nM. *K_i_* calculated from equations **(4)** and **(5)**, describing noncompetitive inhibitors, is 14.9±3.5 nM (n = 3, error bars represent S.D.).
(TIF)

Figure S6 **Progress curves of thrombin inhibitied by EP21 and DV24.** (**A**) Progress curves of thrombin (0.8 nM) inhibited by different concentrations of EP21 using S2238 (100 µM) as substrate, without pre-incubation of thrombin and EP21. The non-linear behavior of the curves at the beginning of the reactions and an improved *IC_50_* with pre-incubation (Figure S4) suggested equilibrium of inhibition was achieved slowly, characteristic of slow-binding inhibitors. (**B**) Progress curves of thrombin (1.65 nM) inhibited by different concentrations of DV24: 0 nM (▪), 0.1 nM (□), 0.3 nM (•), 1 nM (○), 3 nM (▴), 10 nM (▵), 30 nM (▾), 100 nM (▿), 300 nM (⧫) and 1000 nM (⋄) using S2238 (100 µM) as substrate, without pre-incubation of thrombin and DV24. The linear curves indicate the equilibrium of inhibition was achieved upon mixing of thrombin and DV24, characteristic of fast-binding inhibitors.
(TIF)

Figure S7 **Variegin variant DV24 (fast, tight-binding, competitive inhibitor).** (**A**) Dose-response curves of thrombin (1.65 nM) inhibited by DV24 (0.1 nM, 0.3 nM, 1 nM, 3 nM, 10 nM, 30 nM, 100 nM, 300 nM, 1000 nM and 3000 nM) in S2238 (100 µM) showed a right shift with increased pre-incubation time due to cleavage. *IC_50_* are 7.49±0.28 nM without pre-incubation (▪ solid line) and 10.1±0.6 nM with 20 min pre-incubation (○ dotted line) (n = 3, error bars represent S.D.). (**B**) Thrombin (1.65 nM) inhibition was tested with DV24 (0.39 nM, 0.78 nM, 1.56 nM, 3.13 nM, 6.25 nM, 12.5 nM, 25 nM, 50 nM, 100 nM and 200 nM) in S2238 (100 µM) (▪ solid line). Apparent inhibition constant *K_i_′* obtained by fitting data to equation **(2)**, describing fast and tight-binding inhibitor, is 9.74±0.91 nM. *K_i_* calculated from equation **(3)**, describing competitive inhibitors, is 0.306±0.029 nM (n = 3, error bars represent S.D.).
(TIF)

Figure S8 **Variegin variant DV24** ***H12A*** **(fast, tight-binding, competitive inhibitor).** (**A**) Dose-response curves of thrombin (1.65 nM) inhibited by DV24*H12A* (0.001 µM, 0.003 µM, 0.01 µM, 0.03 µM, 0.3 µM, 1 µM, 3 µM, 10 µM and 30 µM) in S2238 (100 µM) showed a right shift with increased pre-incubation time due to cleavage. *IC_50_* are 48.2±12.4 nM without pre-incubation (▪ solid line) and 141±11 nM with 20 min pre-incubation (○ dotted line) (n = 3, error bars represent S.D.). (**B**) Thrombin (1.65 nM) inhibition was tested with DV24*H12A* (1.95 nM, 3.91 nM, 7.81 nM, 15.6 nM, 31.3 nM, 62.5 nM, 125 nM, 250 nM, 500 nM and 1000 nM) in S2238 (100 µM) (▪ solid line). Apparent inhibition constant *K_i_′* obtained by fitting data to equation **(2)**, describing fast and tight-binding inhibitor, is 103±15 nM. *K_i_* calculated from equation **(3)**, describing competitive inhibitors, is 3.23±0.48 nM (n = 3, error bars represent S.D.).
(TIF)

Figure S9 **Variegin variant MH18** ***H12A*** **(fast, noncompetitive inhibitor).** (**A**) Dose-response curves of thrombin (1.65 nM) inhibition by MH18*H12A* (0.001 µM, 0.003 µM, 0.01 µM, 0.03 µM, 0.3 µM, 1 µM, 3 µM, 10 µM and 30 µM) in S2238 (100 µM) are independent of pre-incubation time. *IC_50_* are 328±23 nM without pre-incubation (▪ solid line) and 343±46 nM with 20 min pre-incubation (n = 3, error bars represent S.D.). (**B**) Thrombin (1.65 nM) inhibition was tested with 1 µM MH18*H12A* (○ dotted line) in S2238 (4.69 µM, 9.34 µM, 18.8 µM, 37.5 µM, 75 µM, 150 µM and 300 µM) and without the inhibitor (▪ solid line) in S2238 (3.13 µM, 6.25 µM, 12.5 µM, 25 µM, 50 µM, 100 µM, 200 µM). MH18*H12A* is unable to inhibit thrombin at equimolar concentration, hence is not considered as tight-binding inhibitor. The double-reciprocal plot showed noncompetitive inhibition and *K_i_* is 329±8 nM (n = 3, error bars represent S.D.).
(TIF)

Figure S10 **Variegin variant DV24** ***K10R*** **(fast, tight-binding, competitive inhibitor).** (**A**) Dose-response curves of thrombin (1.65 nM) inhibited by DV24*K10R* (0.1 nM, 0.3 nM, 1 nM, 3 nM, 10 nM, 30 nM, 100 nM, 300 nM, 1000 nM and 3000 nM) in S2238 (100 µM) showed a right shift with increased pre-incubation time due to cleavage. *IC_50_* are 6.98±0.76 nM without pre-incubation (▪ solid line) and 12.0±0.4 nM with 20 min pre-incubation (○ dotted line) (n = 3, error bars represent S.D.). (**B**) Thrombin (1.65 nM) inhibition was tested with DV24*K10R* (0.39 nM, 0.78 nM, 1.56 nM, 3.13 nM, 6.25 nM, 12.5 nM, 25 nM, 50 nM, 100 nM and 200 nM) in S2238 (100 µM) (▪ solid line). Apparent inhibition constant *K_i_′* obtained by fitting data to equation **(2)**, describing fast and tight-binding inhibitor, is 8.27±0.85 nM. *K_i_* calculated from equation **(3)**, describing competitive inhibitors, is 0.259±0.015 nM (n = 3, error bars represent S.D.).
(TIF)

Figure S11 **Variegin variant DV23 (fast, tight-binding, competitive inhibitor).** (**A**) Dose-response curves of thrombin (1.65 nM) inhibited by DV23 (0.1 nM, 0.3 nM, 1 nM, 3 nM, 10 nM, 30 nM, 100 nM, 300 nM, 1000 nM and 3000 nM) in S2238 (100 µM) showed a right shift with increased pre-incubation time due to cleavage. *IC_50_* are 45.4±1.6 nM without pre-incubation (▪ solid line) and 77.8±6.1 nM with 20 min pre-incubation (○ dotted line) (n = 3, error bars represent S.D.). (**B**) Thrombin (1.65 nM) inhibition was tested with DV23 (3.91 nM, 7.81 nM, 15.6 nM, 31.3 nM, 62.5 nM, 125 nM, 250 nM and 500 nM) S2238 (100 µM) (▪ solid line). Apparent inhibition constant *K_i_′* obtained by fitting data to equation **(2)**, describing fast and tight-binding inhibitor, is 69.6±7.8 nM. *K_i_* calculated from equation **(3)**, describing competitive inhibitors, is 2.19±0.23 nM (n = 3, error bars represent S.D.).
(TIF)

Figure S12 **Variegin variant DV23** ***K10R*** **(fast, tight-binding, competitive inhibitor).** (**A**) Dose-response curves of thrombin (1.65 nM) inhibited by DV23*K10R* (0.1 nM, 0.3 nM, 1 nM, 3 nM, 10 nM, 30 nM, 100 nM, 300 nM, 1000 nM and 3000 nM) in S2238 (100 µM) showed a strong right shift with increased pre-incubation time due to cleavage. *IC_50_* are 12.9±1.0 nM without pre-incubation (▪ solid line) and 102±1 nM with 20 min pre-incubation (○ dotted line) (n = 3, error bars represent S.D.). (**B**) Thrombin (1.65 nM) inhibition was tested with DV23*K10R* (3.91 nM, 7.81 nM, 15.6 nM, 31.3 nM, 62.5 nM, 125 nM, 250 nM and 500 nM) in S2238 (100 µM) (▪ solid line). Apparent inhibition constant *K_i_′* obtained by fitting data to equation **(2)**, describing fast and tight-binding inhibitor, is 19.1±1.9 nM. *K_i_* calculated from equation **(3)**, describing competitive inhibitors, is 0.600±0.010 nM (n = 3, error bar represents S.D.).
(TIF)

Figure S13 **Variegin variant EP25** ***A22E*** **(slow binding, competitive inhibitor).** (**A**) Dose-response curves of thrombin (1.65 nM) inhibited by EP25*A22E* (0.1 nM, 0.3 nM, 1 nM, 3 nM, 10 nM, 30 nM, 100 nM, 300 nM, 1000 nM and 3000 nM) in S2238 (100 µM) showed a left shift due to slow binding. *IC_50_* are 124±23 nM without pre-incubation (▪ solid line) and 13.5±2.1 nM with 20 min pre-incubation (○ dotted line) (n = 3, error bars represent S.D.). (**B**) Progress curves (not shown) of thrombin (0.8 nM) inhibited by EP25*A22E* (9.38 nM, 12.5 nM, 18.8 nM, 25 nM, 37.5 nM, 50 nM, 75 nM, 100 nM, 150 nM, 200 nM and 300 nM) in S2238 (100 µM) were fitted to equation **(6)** describing a slow binding inhibitor to obtain a *k* for each concentrations of EP25*A22E*. Plot of *k* against EP25*A22E* concentrations (▪ solid line) is hyperbolic and was fitted to equation **(7)** producing *K_i_′* of 1.02±0.060 nM, representing the dissociation constant of initial collision complex EI (**scheme 1**). *K_i_* calculated from equation **(8)** is 0.311±0.070 nM (n = 3, error bars represent S.D.).
(TIF)

Figure S14 **Variegin variant MH22** ***A22E*** **(fast, tight-binding, noncompetitive inhibitor).** (**A**) Dose-response curves of thrombin (1.65 nM) inhibited by MH22*A22E* (0.1 nM, 0.3 nM, 1 nM, 3 nM, 10 nM, 30 nM, 100 nM, 300 nM, 1000 nM and 3000 nM) in S2238 (100 µM) are independent of pre-incubation time. *IC_50_* are 13.62±0.45 nM without pre-incubation (▪ solid line) and 15.6±0.4 nM with 20 min pre-incubation (○ dotted line) (n = 3, error bars represent S.D.). (**B**) Thrombin (1.65 nM) inhibition was tested with MH22*A22E* (0.39 nM, 0.78 nM, 1.56 nM, 3.13 nM, 6.25 nM, 12.5 nM, 25 nM, 50 nM, 100 nM and 200 nM) in S2238 (100 µM) (▪ solid line). Apparent inhibition constant *K_i_′* obtained by fitting data to equation **(2)**, describing fast and tight-binding inhibitor, is 15.1±1.0 nM. *K_i_* calculated from equations **(4)** and **(5)**, describing noncompetitive inhibitors, is 15.1±1.0 nM (n = 3, error bars represent S.D.).
(TIF)

Figure S15 **Variegin variant DV24** ***Y^phos^*** **(fast, tight-binding, competitive inhibitor).** (**A**) Dose-response curves of thrombin (1.65 nM) inhibited by DV24*Y^phos^* (0.03 nM, 0.1 nM, 0.3 nM, 1 nM, 3 nM, 10 nM, 30 nM, 100 nM, 300 nM and 1000 nM) in S2238 (100 µM) showed a right shift with increased pre-incubation time due to cleavage. *IC_50_* are 8.67±0.45 nM without pre-incubation (▪ solid line) and 12.4±1.2 nM with 20 min pre-incubation (○ dotted line) (n = 3, error bars represent S.D.). (**B**) Thrombin (1.65 nM) inhibition was tested with DV24*Y^phos^* (0.39 nM, 0.78 nM, 1.56 nM, 3.13 nM, 6.25 nM, 12.5 nM, 25 nM, 50 nM, 100 nM and 200 nM) in S2238 (100 µM) (▪ solid line). Apparent inhibition constant *K_i_′* obtained by fitting data to equation **(2)**, describing fast and tight-binding inhibitors, is 10.4±1.0 nM. *K_i_* calculated from equation **(3)**, describing competitive inhibitors, the inhibition constant is 0.327±0.032 nM (n = 3, error bars represent S.D.).
(TIF)

Figure S16 **Variegin variant DV24** ***K10RY^phos^*** **(fast, tight-binding, competitive inhibitor).** (**A**) Dose-response curves of thrombin (1.65 nM) inhibited by DV24*K10RY^phos^* (0.03 nM, 0.1 nM, 0.3 nM, 1 nM, 3 nM, 10 nM, 30 nM, 100 nM, 300 nM and 1000 nM) in S2238 (100 µM) showed a right shift with increased pre-incubation time due to cleavage. *IC_50_* are 4.64±0.78 nM without pre-incubation (▪ solid line) and 7.80±1.80 nM with 20 min pre-incubation (○ dotted line) (n = 3, error bars represent S.D.). (**B**) Thrombin (1.65 nM) inhibition was tested with DV24*K10RY^phos^* (0.39 nM, 0.78 nM, 1.56 nM, 3.13 nM, 6.25 nM, 12.5 nM, 25 nM, 50 nM, 100 nM and 200 nM) in S2238 (100 µM) (▪ solid line). Apparent inhibition constant *K_i_′* obtained by fitting data to equation **(2)**, describing fast and tight-binding inhibitors, is 4.78±0.57 nM. *K_i_* calculated from equation **(3)**, describing competitive inhibitors, is 0.150±0.018 nM (n = 3, error bars represent S.D.).
(TIF)

Figure S17 **Variegin variant DV24** ***Y^sulf^*** **(fast, tight-binding, competitive inhibitor).** (**A**) Dose-response curves of thrombin (1.65 nM) inhibited by DV24*Y^sulf^* (0.05 nM, 0.15 nM, 0.45 nM, 1.5 nM, 4.5 nM, 15 nM, 45 nM, 150 nM, 450 nM and 1500 nM) in S2238 (100 µM) showed a right shift with increased pre-incubation time due to cleavage. *IC_50_* are 1.66±0.18 nM without pre-incubation (▪ solid line) and 2.02±0.29 nM with 20 min pre-incubation (○ dotted line) (n = 3, error bars represent S.D.). (**B**) Thrombin (1.65 nM) inhibition was tested with DV24*Y^sulf^* (0.20 nM, 0.39 nM, 0.78 nM, 1.56 nM, 3.13 nM, 6.25 nM, 12.5 nM, 25 nM, 50 nM and 100 nM) in S2238 (100 µM) (▪ solid line). Apparent inhibition constant *K_i_′* obtained by fitting data to equation **(2)**, describing fast and tight-binding inhibitor, is 1.78±0.47 nM. *K_i_* calculated from equation **(3)**, describing competitive inhibitors, is 0.056±0.015 nM (n = 3, error bars represent S.D.).
(TIF)

Figure S18 **Variegin variant DV24** ***K10RY^sulf^*** **(fast, tight-binding, competitive inhibitor).** (**A**) Dose-response curves of thrombin (1.65 nM) inhibited by DV24*K10RY^sulf^* (0.05 nM, 0.15 nM, 0.45 nM, 1.5 nM, 4.5 nM, 15 nM, 45 nM, 150 nM, 450 nM and 1500 nM) in S2238 (100 µM) showed a right shift with increased pre-incubation time due to cleavage. *IC_50_* are 1.39±0.17 nM without pre-incubation (▪ solid line) and 1.66±0.21 nM with 20 min pre-incubation (○ dotted line) (n = 3, error bars represent S.D.). (**B**) Thrombin (1.65 nM) inhibition was tested with DV24*K10RY^sulf^* (0.20 nM, 0.39 nM, 0.78 nM, 1.56 nM, 3.13 nM, 6.25 nM, 12.5 nM, 25 nM, 50 nM and 100 nM) in S2238 (100 µM) (▪ solid line). Apparent inhibition constant *K_i_′* obtained by fitting data to equation **(2)**, describing fast and tight-binding inhibitors, is 1.33±0.19 nM. *K_i_* calculated from equation **(3)**, describing competitive inhibitors, is 0.0420±0.0061 nM (n = 3, error bars represent S.D.).
(TIF)

Figure S19 **Variegin variant MH18** ***Y^sulf^*** **(fast, tight-binding, noncompetitive inhibitor).** (**A**) Dose-response curves of thrombin (1.65 nM) inhibited by MH18*Y^sulf^* (0.03 nM, 0.1 nM, 0.3 nM, 1 nM, 3 nM, 10 nM, 30 nM, 100 nM, 300 nM and 1000 nM) in S2238 (100 µM) are independent of pre-incubation time. *IC_50_* are 1.26±0.18 nM without pre-incubation (▪ solid line) and 1.17±0.14 nM with 20 min pre-incubation (○ dotted line) (n = 3, error bar represents S.D.). (**B**) Thrombin (1.65 nM) inhibition was tested with MH18*Y^sulf^* (0.20 nM, 0.39 nM, 0.78 nM, 1.56 nM, 3.13 nM, 6.25 nM, 12.5 nM, 25 nM, 50 nM and 100 nM) in S2238 (100 µM) (▪ solid line). Apparent inhibition constant *K_i_′* obtained by fitting data to equation **(2)**, describing fast and tight-binding inhibitors, is 1.25±0.18 nM. *K_i_* calculated from equations **(4)** and **(5)**, describing noncompetitive inhibitors, is 1.25±0.18 nM (n = 3, error bar represents S.D.).
(TIF)

Figure S20 **Noncompetitive inhibition of thrombin by MH22.** s-Variegin binds to both the non-prime and prime subsites of thrombin active site and is cleaved between Lys-Met. After cleavage, the fragment C-terminal to the scissile bond (MH22) noncompetitively inhibits thrombin. The chromogenic substrate S2238 binds mainly to the non-prime subsites and is cleaved between Arg and *para*-nitroaniline (*p*NA). The overlaps between s-variegin and S2238 binding sites resulted in the observed competitive inhibition. In contrast, the noncompetitive inhibition observed for MH22 showed the lack of overlaps between MH22 and S2238 even in the S1′ subsite (red box). Indeed, no density was observed for P1′ Met in the present structure, most likely reflects the lack of contact with thrombin and hence leaves a free S1′ site for the binding of *p*NA moiety when MH22 is bound to thrombin.
(TIF)

Table S1 A list of possible direct hydrogen bonds between s-variegin and thrombin calculated based on the online server PISA [28].
(TIF)

Materials and Methods S1 A detailed account for the selection and use of equations to fit the data of thrombin inhibitions is available in Materials and Methods S1.
(DOC)
