# Supplementary figures and images for: Crystal Structure of Thrombin in Complex with S-Variegin: Insights of a Novel Mechanism of Inhibition and Design of Tunable Thrombin Inhibitors
Source: PLoS One. 2011 Oct 28;6(10):e26367. doi: 10.1371/journal.pone.0026367 (PMC3203879; doi:10.1371/journal.pone.0026367)

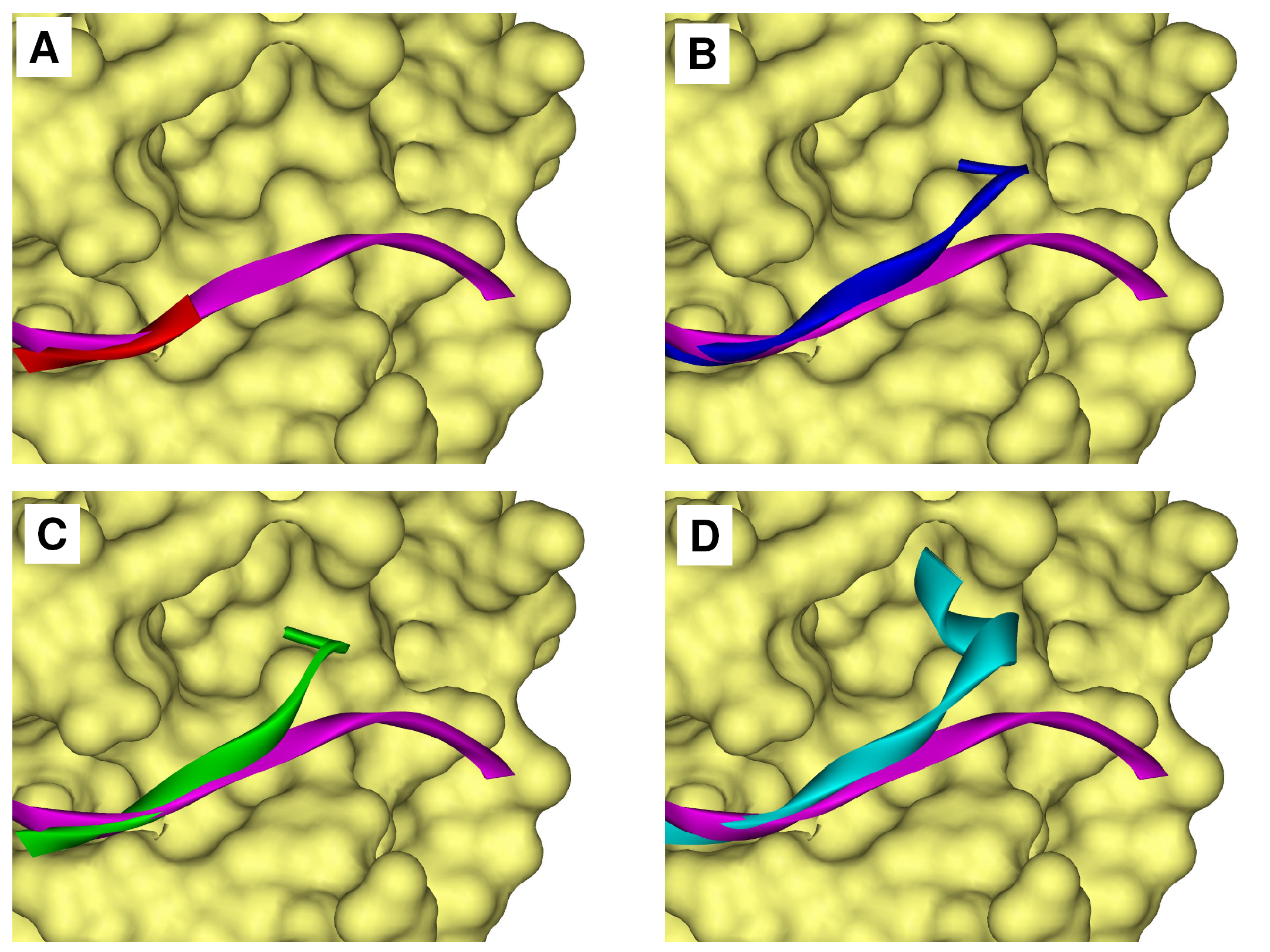

Supplement: Figure S1 — Conformation of s-variegin C-terminus. s-Variegin C-terminus (pink) has a vastly different conformation compared to hirulog-1, hirulog-3, hirugen and sulfo-hirudin: (A) Residues PEEYL in hirulog-1 (red) are disordered and missing from the structure. (B) Residues PEEYL in hirulog-3 (blue) form a 310 helix turn. (C) These residues in hirugen (green), with sulfated tyrosine, also form a 310 helix turn. (D) Other than Tyr-sulfation, sulfo-hirudin (cyan) C-terminus has an extra Gln, forms a full α-helical turn. (TIF) [file pone.0026367.s001.tif]

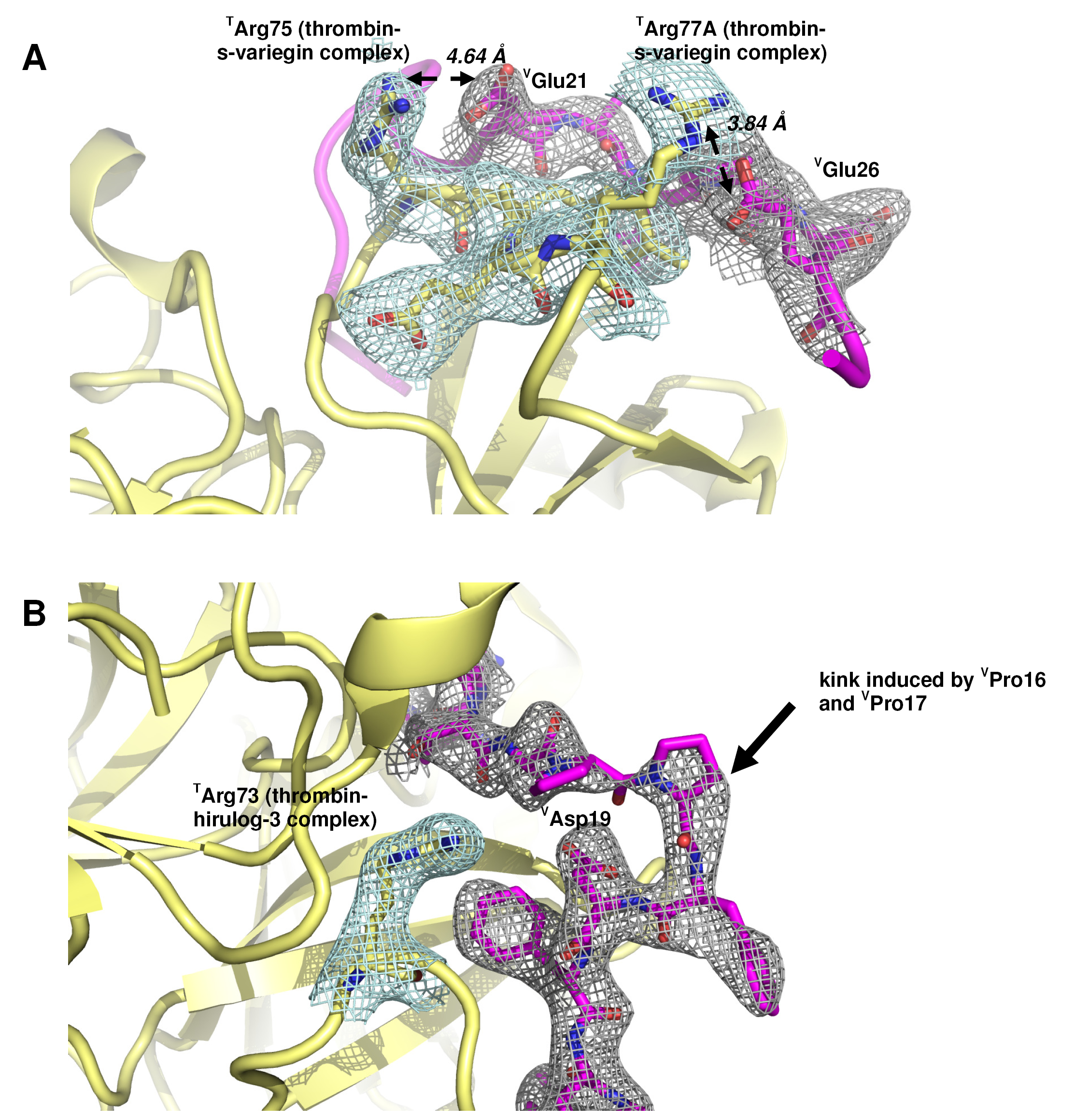

Supplement: Figure S2 — Electrostatic interactions in thrombin:s-variegin structure. (A) Figure shows the electron density map (2Fo-Fc, 0.9σ) of residues described in Figure 4A in the main manuscript. Thrombin is colored yellow and s-variegin is colored pink. Map for thrombin colored in light cyan and map for s-variegin colored in gray. Residues involved in forming salt bridges are labeled. (B) Figure shows the electron density map (2Fo-Fc, 0.9σ) of residues described in Figure 4B in the main manuscript. Thrombin is colored yellow and s-variegin is colored pink. Map for thrombin colored in light cyan and map for s-variegin colored in gray. (TIF) [file pone.0026367.s002.tif]

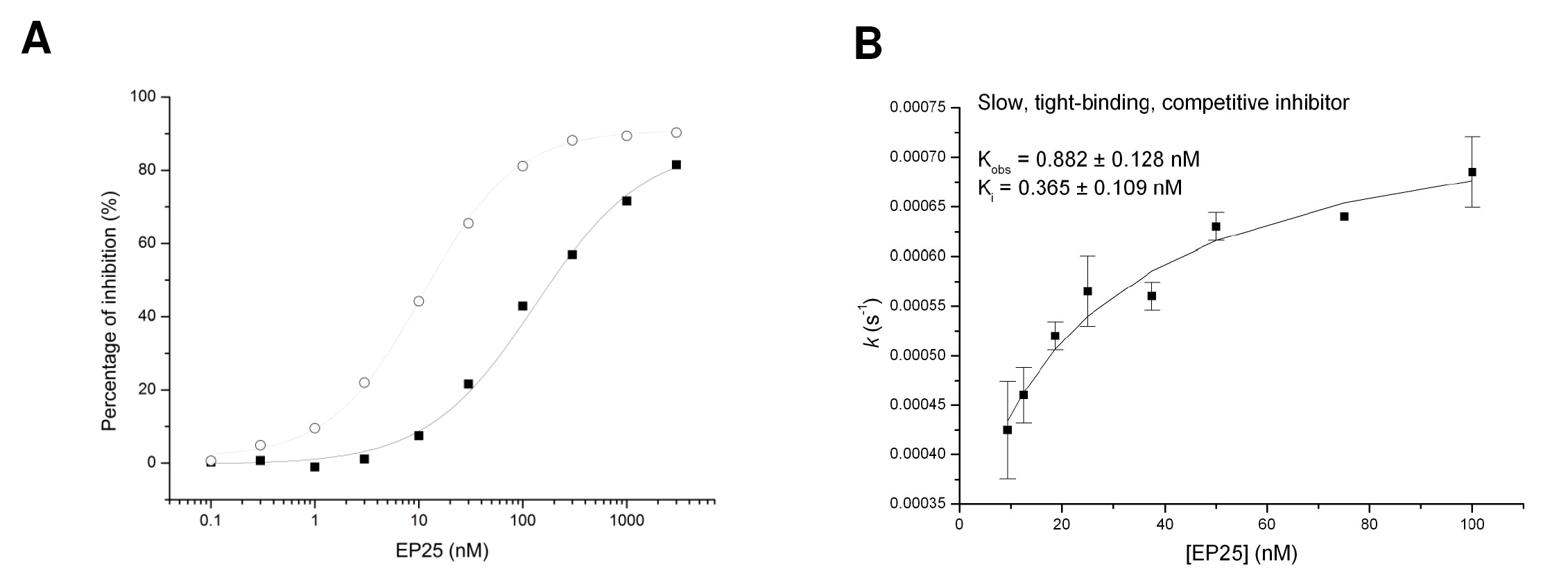

Supplement: Figure S3 — Variegin variant EP25 (slow binding, competitive inhibitor). (A) Dose response curves of thrombin (1.65 nM) inhibited by EP25 (0.1 nM, 0.3 nM, 1 nM, 3 nM, 10 nM, 30 nM, 100 nM, 300 nM, 1000 nM, 3000 nM) in S2238 (100 µM) showed a left shift with increased pre-incubation time due to slow binding. IC50 are 173±26 nM without pre-incubation (▪ solid line) and 13.1±0.7 nM with 20 min pre-incubation (○ dotted line) (n = 3, error bars represent S.D.). (B) Progress curves (not shown) of thrombin (0.8 nM) inhibited by EP25 (9.4 nM, 12.5 nM, 18.8 nM, 25 nM, 37.5 nM, 50 nM, 75 nM and 100 nM) in S2238 (100 µM) were fitted to equation (6) describing a slow binding inhibitor to obtain a k for each concentrations of EP25. Plot of k against EP25 concentrations (▪ solid line) is hyperbolic and fitted to equation (7) producing Ki′ of 0.882±0.128 nM, representing the dissociation constant of initial collision complex EI (scheme 1). Ki calculated from equation (8) is 0.365±0.109 nM (n = 3, error bars represent S.D.). (TIF) [file pone.0026367.s003.tif]

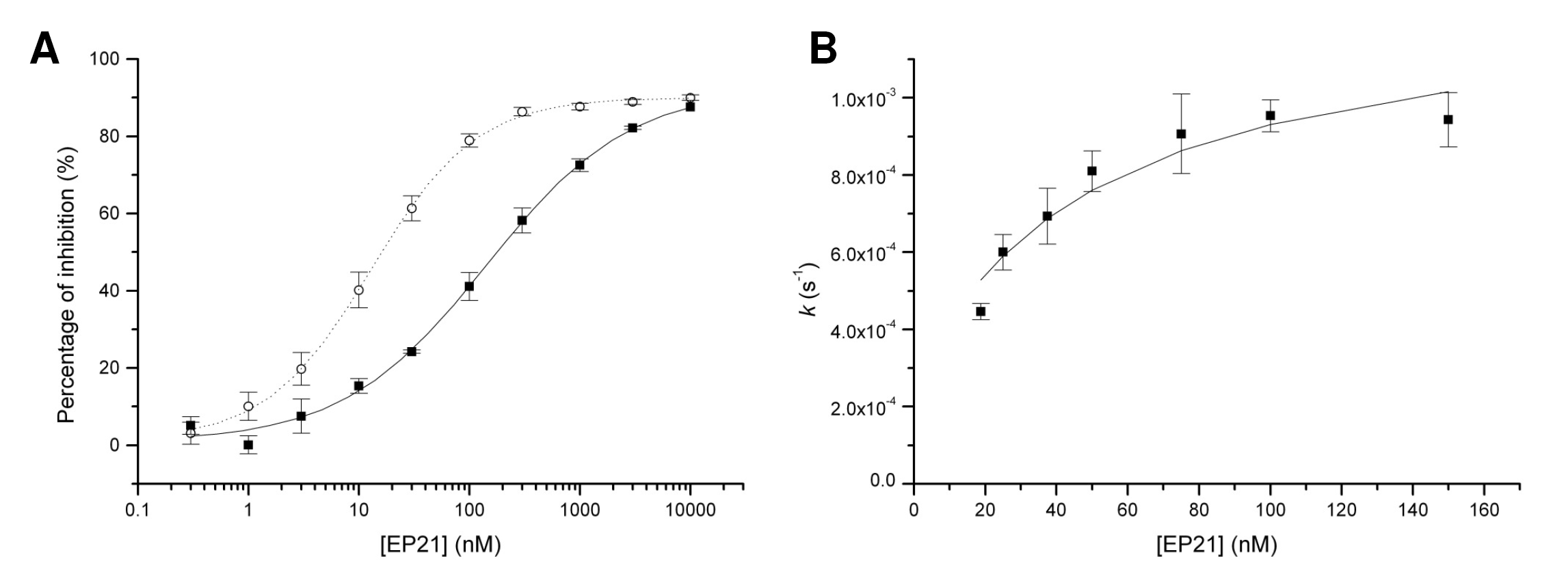

Supplement: Figure S4 — Variegin variant EP21 (slow binding, competitive inhibitor). (A) Dose response curves of thrombin (1.65 nM) inhibited by EP21 (0.3 nM, 1 nM, 3 nM, 10 nM, 30 nM, 100 nM, 300 nM, 1000 nM, 3000 nM and 10000 nM) in S2238 (100 µM) showed a left shift with increased pre-incubation time due to slow binding. IC50 are 177±7 nM without pre-incubation (▪ solid line) and 16.2±2.9 nM with 20 min pre-incubation (○ dotted line) (n = 3, error bars represent S.D.). (B) Progress curves (Figure S4) of thrombin (0.8 nM) inhibited by EP21 (18.8 nM, 25 nM, 37.5 nM, 50 nM, 75 nM, 100 nM and 150 nM) in S2238 (100 µM) were fitted to equation (6) describing a slow binding inhibitor to obtain a k for each concentrations of EP21. Plot of k against EP21 concentrations (▪ solid line) is hyperbolic and fitted to equation (7) producing Ki′ of 1.66±0.36 nM, representing the dissociation constant of initial collision complex EI (scheme 1). Ki calculated from equation (8) is 0.315±0.024 nM (n = 3, error bars represent S.D.). (TIF) [file pone.0026367.s004.tif]

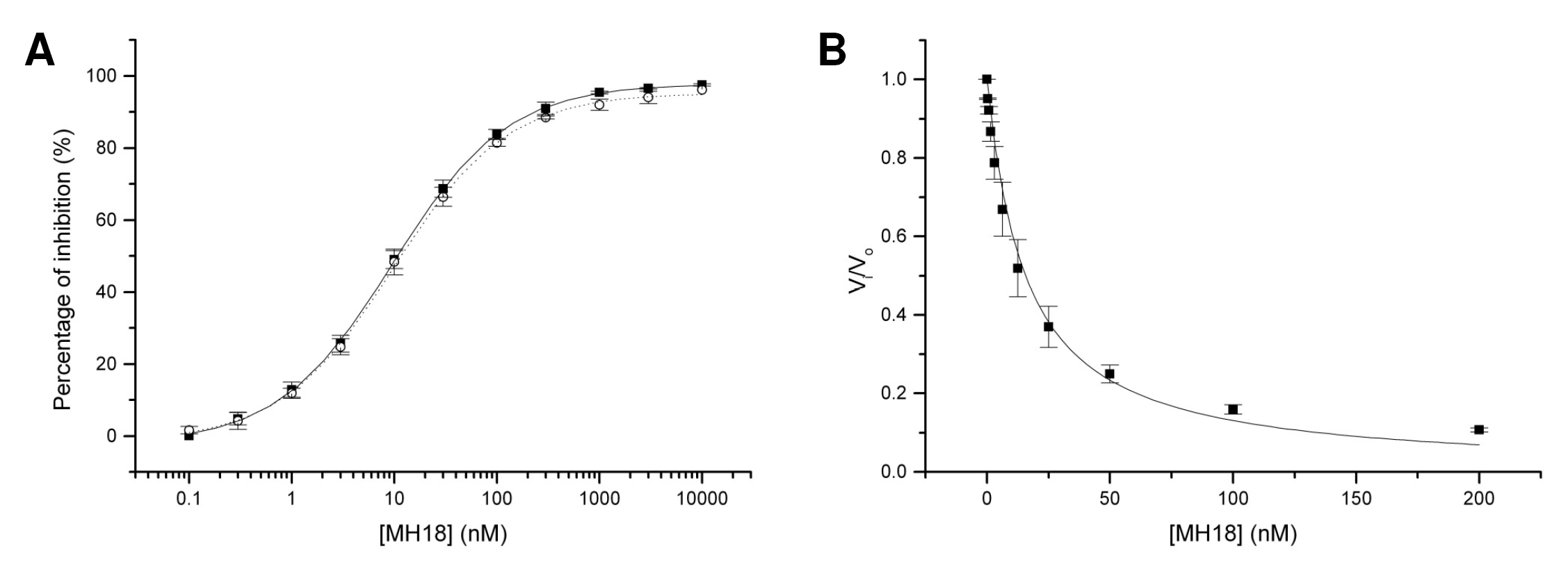

Supplement: Figure S5 — Variegin variant MH18 (fast, tight-binding, noncompetitive inhibitor). (A) Dose response curves of thrombin inhibition (1.65 nM) by MH18 (0.1 nM, 0.3 nM, 1 nM, 3 nM, 10 nM, 30 nM, 100 nM, 300 nM, 1000 nM, 3000 nM and 10000 nM) in S2238 (100 µM) are independent of pre-incubation time. IC50 are 10.9±1.2 nM without pre-incubation (▪ solid line) and 11.7±1.9 nM with 20 min pre-incubation (○ dotted line) (n = 3, error bars represent S.D.). (B) Thrombin (1.65 nM) inhibition was tested with MH18 (0.39 nM, 0.78 nM, 1.56 nM, 3.13 nM, 6.25 nM, 12.5 nM, 25 nM, 50 nM, 100 nM and 200 nM) in S2238 (100 µM) (▪ solid line). Apparent inhibition constant Ki′ obtained by fitting data to equation (2), describing fast and tight-binding inhibitor, is 14.9±3.5 nM. Ki calculated from equations (4) and (5), describing noncompetitive inhibitors, is 14.9±3.5 nM (n = 3, error bars represent S.D.). (TIF) [file pone.0026367.s005.tif]

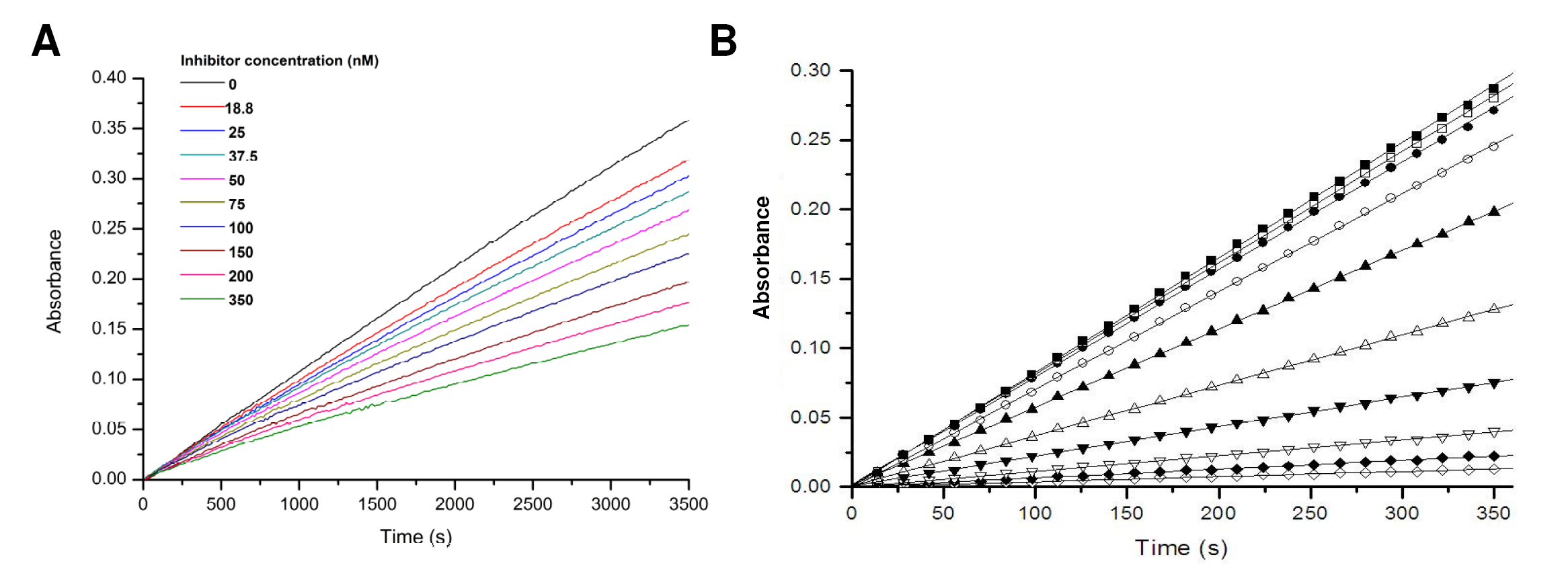

Supplement: Figure S6 — Progress curves of thrombin inhibitied by EP21 and DV24. (A) Progress curves of thrombin (0.8 nM) inhibited by different concentrations of EP21 using S2238 (100 µM) as substrate, without pre-incubation of thrombin and EP21. The non-linear behavior of the curves at the beginning of the reactions and an improved IC50 with pre-incubation (Figure S4) suggested equilibrium of inhibition was achieved slowly, characteristic of slow-binding inhibitors. (B) Progress curves of thrombin (1.65 nM) inhibited by different concentrations of DV24: 0 nM (▪), 0.1 nM (□), 0.3 nM (•), 1 nM (○), 3 nM (▴), 10 nM (▵), 30 nM (▾), 100 nM (▿), 300 nM (⧫) and 1000 nM (⋄) using S2238 (100 µM) as substrate, without pre-incubation of thrombin and DV24. The linear curves indicate the equilibrium of inhibition was achieved upon mixing of thrombin and DV24, characteristic of fast-binding inhibitors. (TIF) [file pone.0026367.s006.tif]

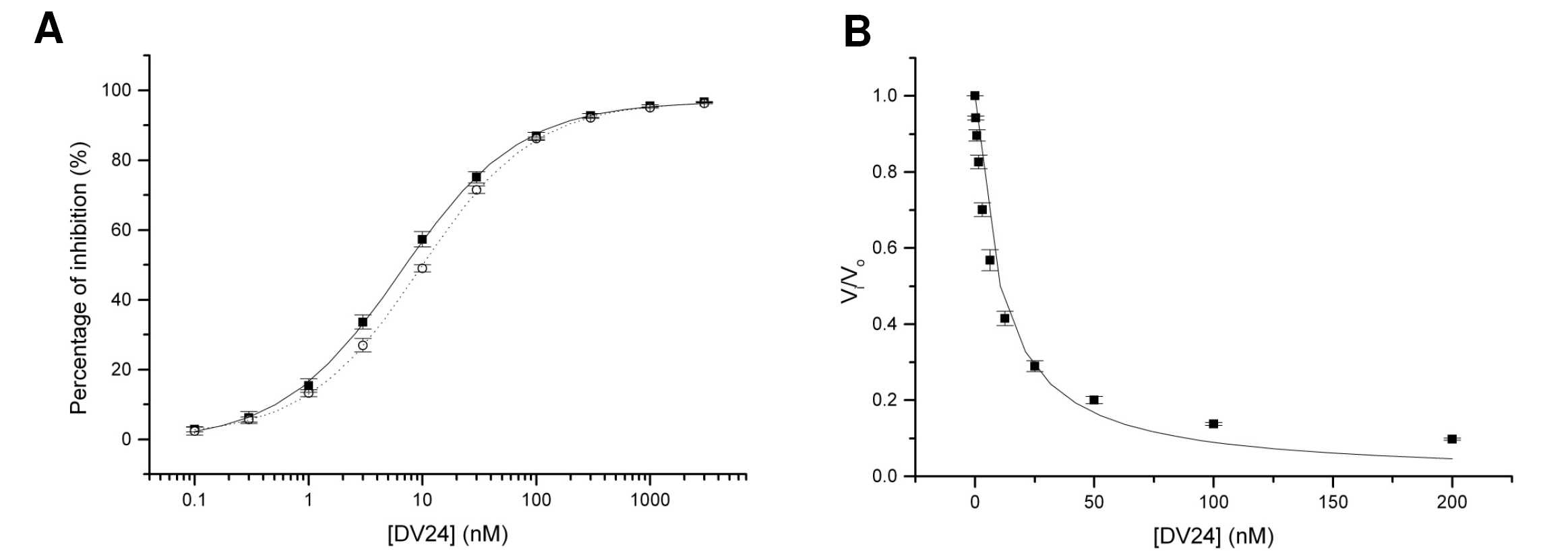

Supplement: Figure S7 — Variegin variant DV24 (fast, tight-binding, competitive inhibitor). (A) Dose-response curves of thrombin (1.65 nM) inhibited by DV24 (0.1 nM, 0.3 nM, 1 nM, 3 nM, 10 nM, 30 nM, 100 nM, 300 nM, 1000 nM and 3000 nM) in S2238 (100 µM) showed a right shift with increased pre-incubation time due to cleavage. IC50 are 7.49±0.28 nM without pre-incubation (▪ solid line) and 10.1±0.6 nM with 20 min pre-incubation (○ dotted line) (n = 3, error bars represent S.D.). (B) Thrombin (1.65 nM) inhibition was tested with DV24 (0.39 nM, 0.78 nM, 1.56 nM, 3.13 nM, 6.25 nM, 12.5 nM, 25 nM, 50 nM, 100 nM and 200 nM) in S2238 (100 µM) (▪ solid line). Apparent inhibition constant Ki′ obtained by fitting data to equation (2), describing fast and tight-binding inhibitor, is 9.74±0.91 nM. Ki calculated from equation (3), describing competitive inhibitors, is 0.306±0.029 nM (n = 3, error bars represent S.D.). (TIF) [file pone.0026367.s007.tif]

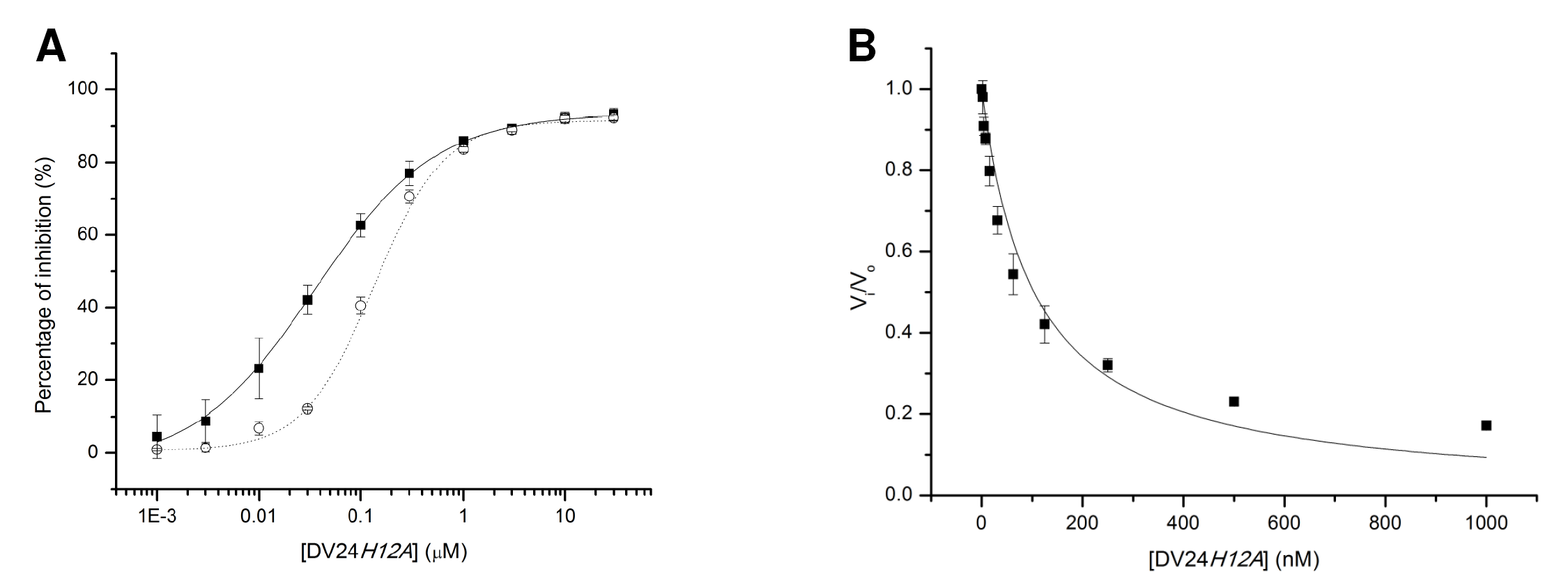

Supplement: Figure S8 — Variegin variant DV24 H12A (fast, tight-binding, competitive inhibitor). (A) Dose-response curves of thrombin (1.65 nM) inhibited by DV24H12A (0.001 µM, 0.003 µM, 0.01 µM, 0.03 µM, 0.3 µM, 1 µM, 3 µM, 10 µM and 30 µM) in S2238 (100 µM) showed a right shift with increased pre-incubation time due to cleavage. IC50 are 48.2±12.4 nM without pre-incubation (▪ solid line) and 141±11 nM with 20 min pre-incubation (○ dotted line) (n = 3, error bars represent S.D.). (B) Thrombin (1.65 nM) inhibition was tested with DV24H12A (1.95 nM, 3.91 nM, 7.81 nM, 15.6 nM, 31.3 nM, 62.5 nM, 125 nM, 250 nM, 500 nM and 1000 nM) in S2238 (100 µM) (▪ solid line). Apparent inhibition constant Ki′ obtained by fitting data to equation (2), describing fast and tight-binding inhibitor, is 103±15 nM. Ki calculated from equation (3), describing competitive inhibitors, is 3.23±0.48 nM (n = 3, error bars represent S.D.). (TIF) [file pone.0026367.s008.tif]

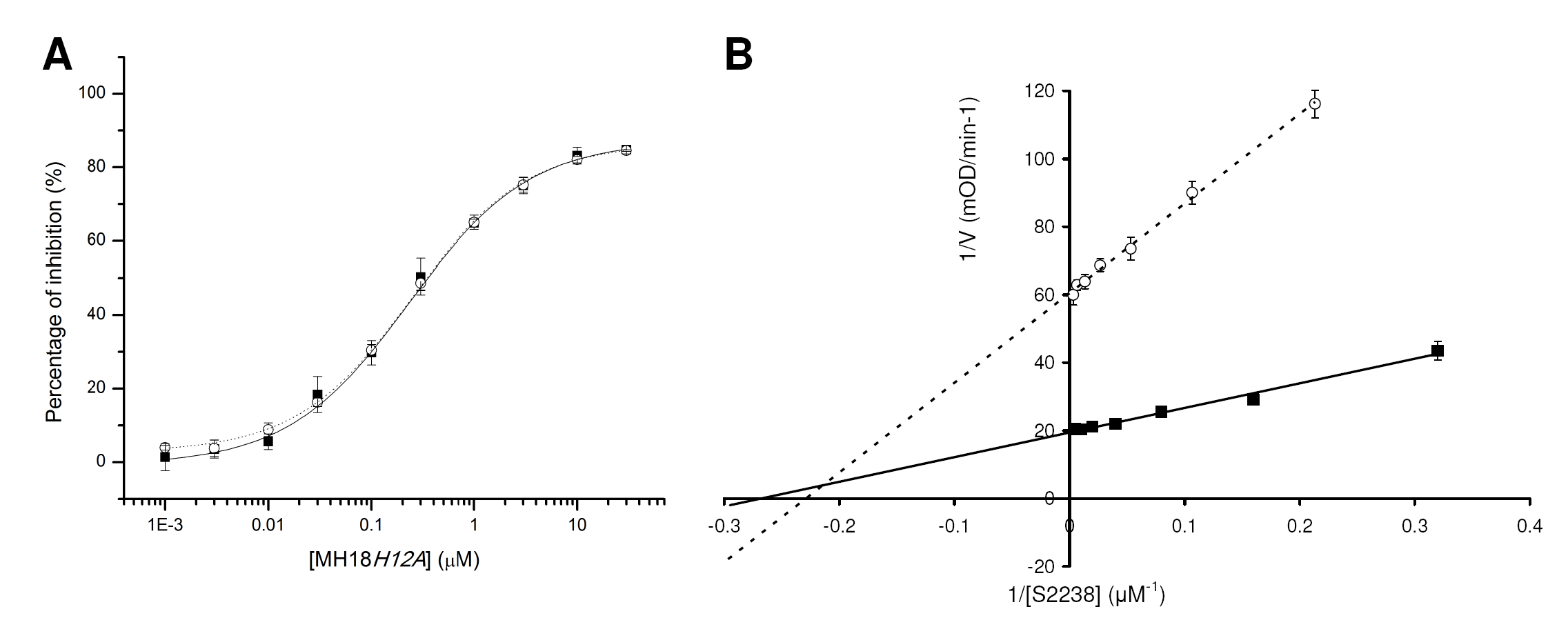

Supplement: Figure S9 — Variegin variant MH18 H12A (fast, noncompetitive inhibitor). (A) Dose-response curves of thrombin (1.65 nM) inhibition by MH18H12A (0.001 µM, 0.003 µM, 0.01 µM, 0.03 µM, 0.3 µM, 1 µM, 3 µM, 10 µM and 30 µM) in S2238 (100 µM) are independent of pre-incubation time. IC50 are 328±23 nM without pre-incubation (▪ solid line) and 343±46 nM with 20 min pre-incubation (n = 3, error bars represent S.D.). (B) Thrombin (1.65 nM) inhibition was tested with 1 µM MH18H12A (○ dotted line) in S2238 (4.69 µM, 9.34 µM, 18.8 µM, 37.5 µM, 75 µM, 150 µM and 300 µM) and without the inhibitor (▪ solid line) in S2238 (3.13 µM, 6.25 µM, 12.5 µM, 25 µM, 50 µM, 100 µM, 200 µM). MH18H12A is unable to inhibit thrombin at equimolar concentration, hence is not considered as tight-binding inhibitor. The double-reciprocal plot showed noncompetitive inhibition and Ki is 329±8 nM (n = 3, error bars represent S.D.). (TIF) [file pone.0026367.s009.tif]

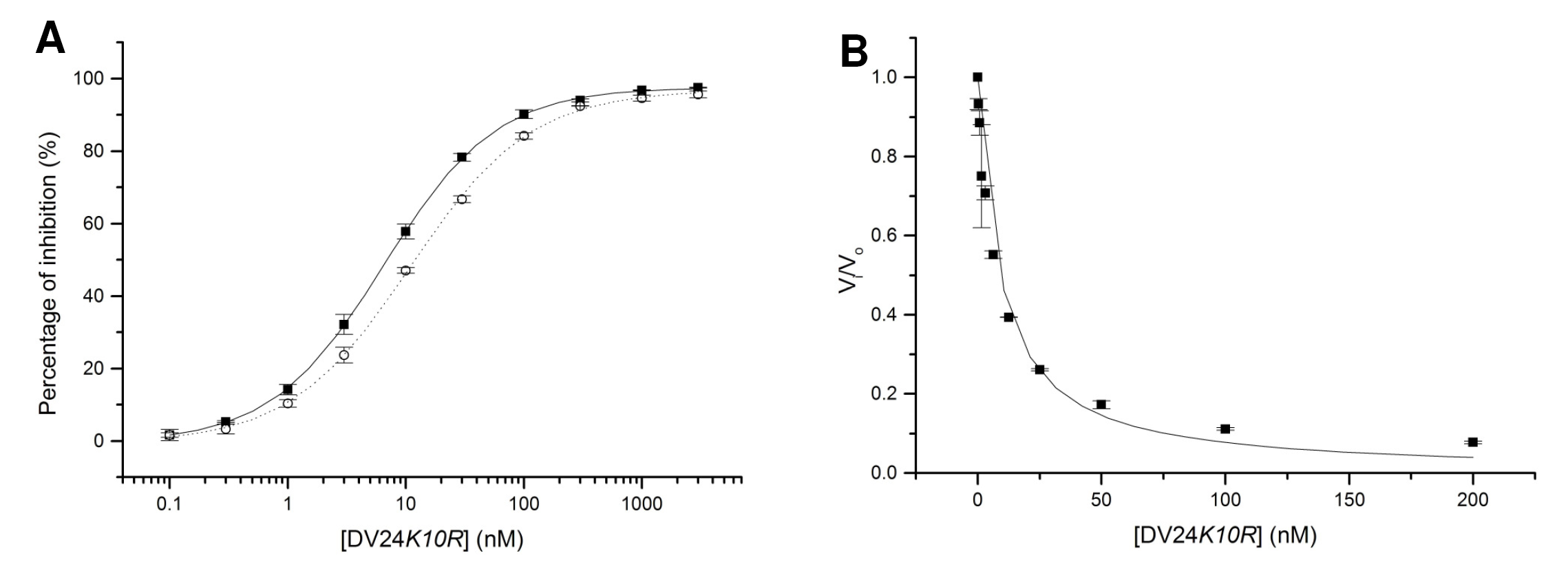

Supplement: Figure S10 — Variegin variant DV24 K10R (fast, tight-binding, competitive inhibitor). (A) Dose-response curves of thrombin (1.65 nM) inhibited by DV24K10R (0.1 nM, 0.3 nM, 1 nM, 3 nM, 10 nM, 30 nM, 100 nM, 300 nM, 1000 nM and 3000 nM) in S2238 (100 µM) showed a right shift with increased pre-incubation time due to cleavage. IC50 are 6.98±0.76 nM without pre-incubation (▪ solid line) and 12.0±0.4 nM with 20 min pre-incubation (○ dotted line) (n = 3, error bars represent S.D.). (B) Thrombin (1.65 nM) inhibition was tested with DV24K10R (0.39 nM, 0.78 nM, 1.56 nM, 3.13 nM, 6.25 nM, 12.5 nM, 25 nM, 50 nM, 100 nM and 200 nM) in S2238 (100 µM) (▪ solid line). Apparent inhibition constant Ki′ obtained by fitting data to equation (2), describing fast and tight-binding inhibitor, is 8.27±0.85 nM. Ki calculated from equation (3), describing competitive inhibitors, is 0.259±0.015 nM (n = 3, error bars represent S.D.). (TIF) [file pone.0026367.s010.tif]

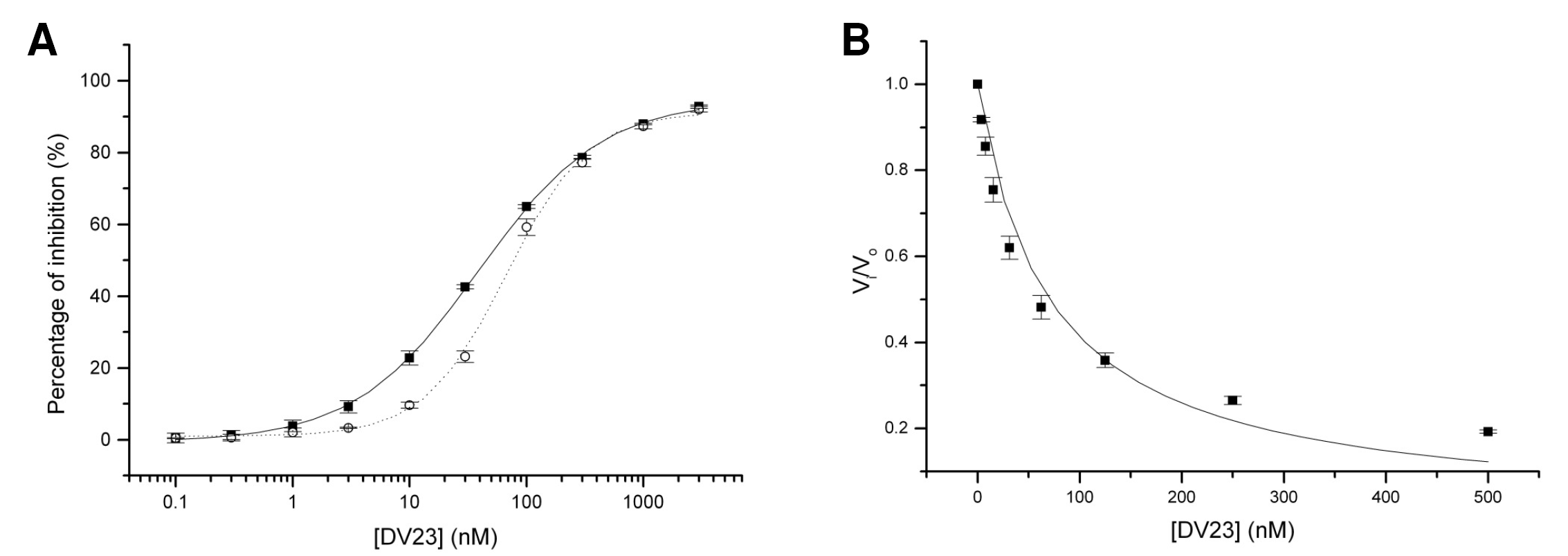

Supplement: Figure S11 — Variegin variant DV23 (fast, tight-binding, competitive inhibitor). (A) Dose-response curves of thrombin (1.65 nM) inhibited by DV23 (0.1 nM, 0.3 nM, 1 nM, 3 nM, 10 nM, 30 nM, 100 nM, 300 nM, 1000 nM and 3000 nM) in S2238 (100 µM) showed a right shift with increased pre-incubation time due to cleavage. IC50 are 45.4±1.6 nM without pre-incubation (▪ solid line) and 77.8±6.1 nM with 20 min pre-incubation (○ dotted line) (n = 3, error bars represent S.D.). (B) Thrombin (1.65 nM) inhibition was tested with DV23 (3.91 nM, 7.81 nM, 15.6 nM, 31.3 nM, 62.5 nM, 125 nM, 250 nM and 500 nM) S2238 (100 µM) (▪ solid line). Apparent inhibition constant Ki′ obtained by fitting data to equation (2), describing fast and tight-binding inhibitor, is 69.6±7.8 nM. Ki calculated from equation (3), describing competitive inhibitors, is 2.19±0.23 nM (n = 3, error bars represent S.D.). (TIF) [file pone.0026367.s011.tif]

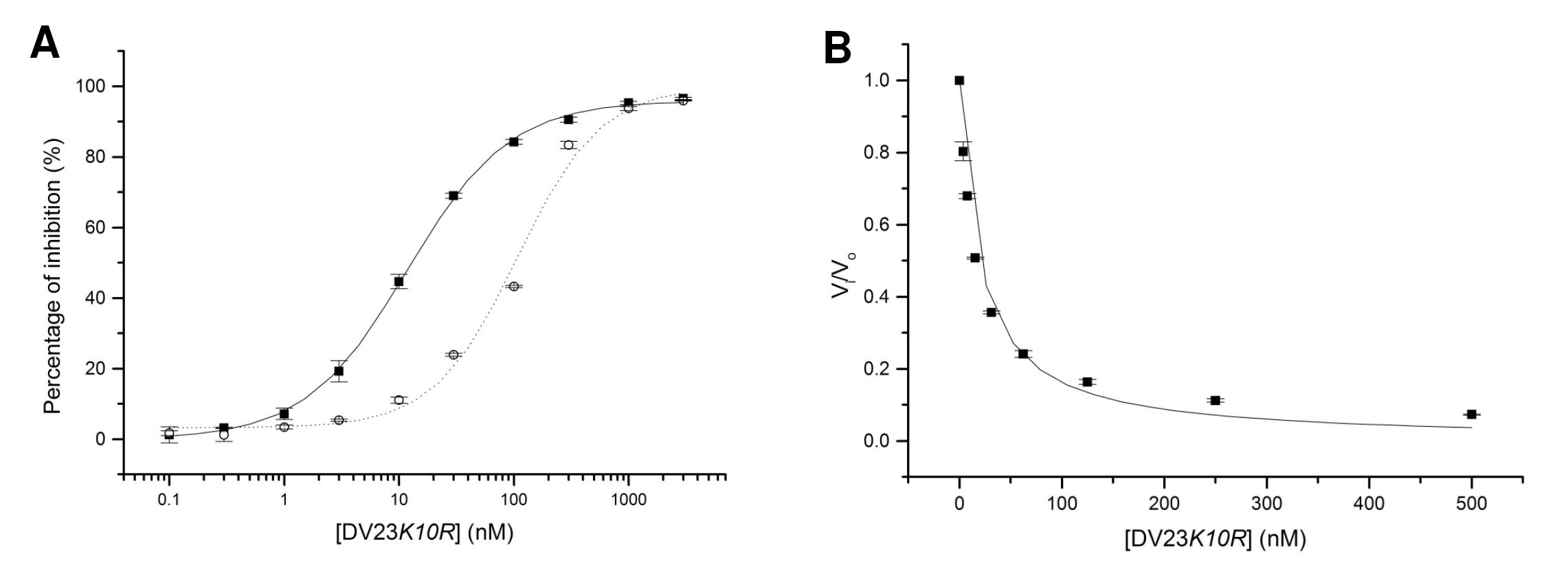

Supplement: Figure S12 — Variegin variant DV23 K10R (fast, tight-binding, competitive inhibitor). (A) Dose-response curves of thrombin (1.65 nM) inhibited by DV23K10R (0.1 nM, 0.3 nM, 1 nM, 3 nM, 10 nM, 30 nM, 100 nM, 300 nM, 1000 nM and 3000 nM) in S2238 (100 µM) showed a strong right shift with increased pre-incubation time due to cleavage. IC50 are 12.9±1.0 nM without pre-incubation (▪ solid line) and 102±1 nM with 20 min pre-incubation (○ dotted line) (n = 3, error bars represent S.D.). (B) Thrombin (1.65 nM) inhibition was tested with DV23K10R (3.91 nM, 7.81 nM, 15.6 nM, 31.3 nM, 62.5 nM, 125 nM, 250 nM and 500 nM) in S2238 (100 µM) (▪ solid line). Apparent inhibition constant Ki′ obtained by fitting data to equation (2), describing fast and tight-binding inhibitor, is 19.1±1.9 nM. Ki calculated from equation (3), describing competitive inhibitors, is 0.600±0.010 nM (n = 3, error bar represents S.D.). (TIF) [file pone.0026367.s012.tif]

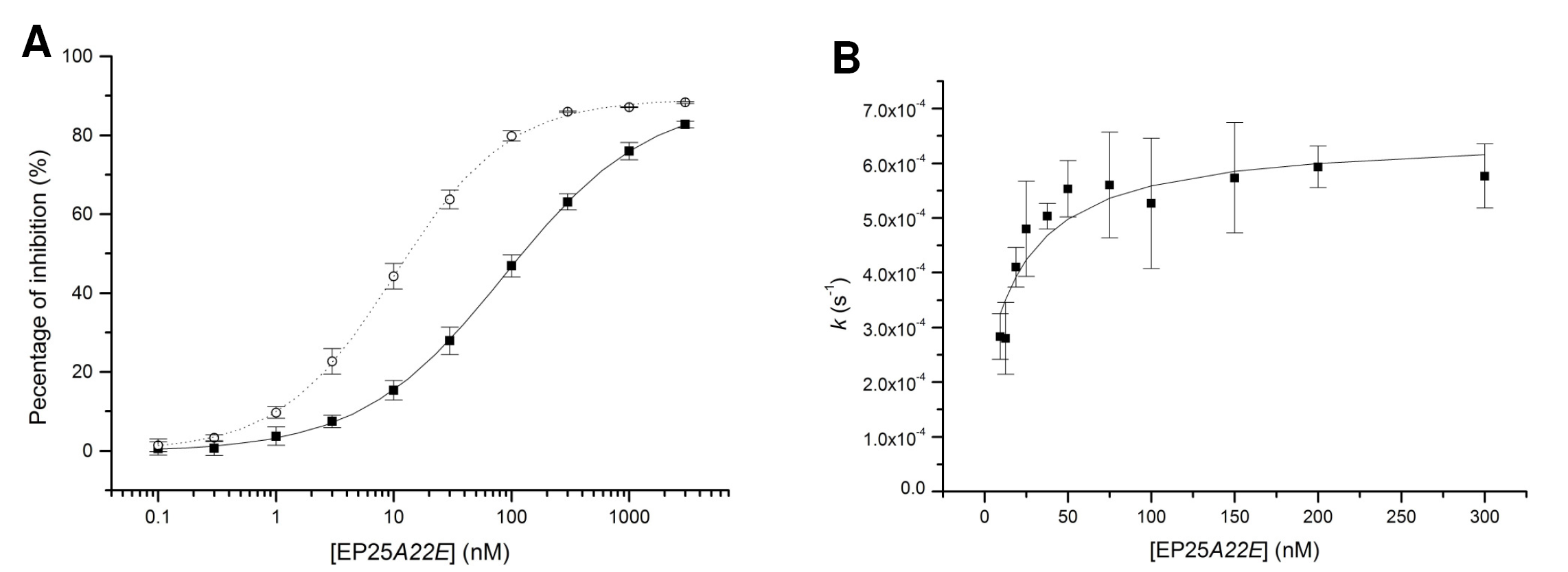

Supplement: Figure S13 — Variegin variant EP25 A22E (slow binding, competitive inhibitor). (A) Dose-response curves of thrombin (1.65 nM) inhibited by EP25A22E (0.1 nM, 0.3 nM, 1 nM, 3 nM, 10 nM, 30 nM, 100 nM, 300 nM, 1000 nM and 3000 nM) in S2238 (100 µM) showed a left shift due to slow binding. IC50 are 124±23 nM without pre-incubation (▪ solid line) and 13.5±2.1 nM with 20 min pre-incubation (○ dotted line) (n = 3, error bars represent S.D.). (B) Progress curves (not shown) of thrombin (0.8 nM) inhibited by EP25A22E (9.38 nM, 12.5 nM, 18.8 nM, 25 nM, 37.5 nM, 50 nM, 75 nM, 100 nM, 150 nM, 200 nM and 300 nM) in S2238 (100 µM) were fitted to equation (6) describing a slow binding inhibitor to obtain a k for each concentrations of EP25A22E. Plot of k against EP25A22E concentrations (▪ solid line) is hyperbolic and was fitted to equation (7) producing Ki′ of 1.02±0.060 nM, representing the dissociation constant of initial collision complex EI (scheme 1). Ki calculated from equation (8) is 0.311±0.070 nM (n = 3, error bars represent S.D.). (TIF) [file pone.0026367.s013.tif]

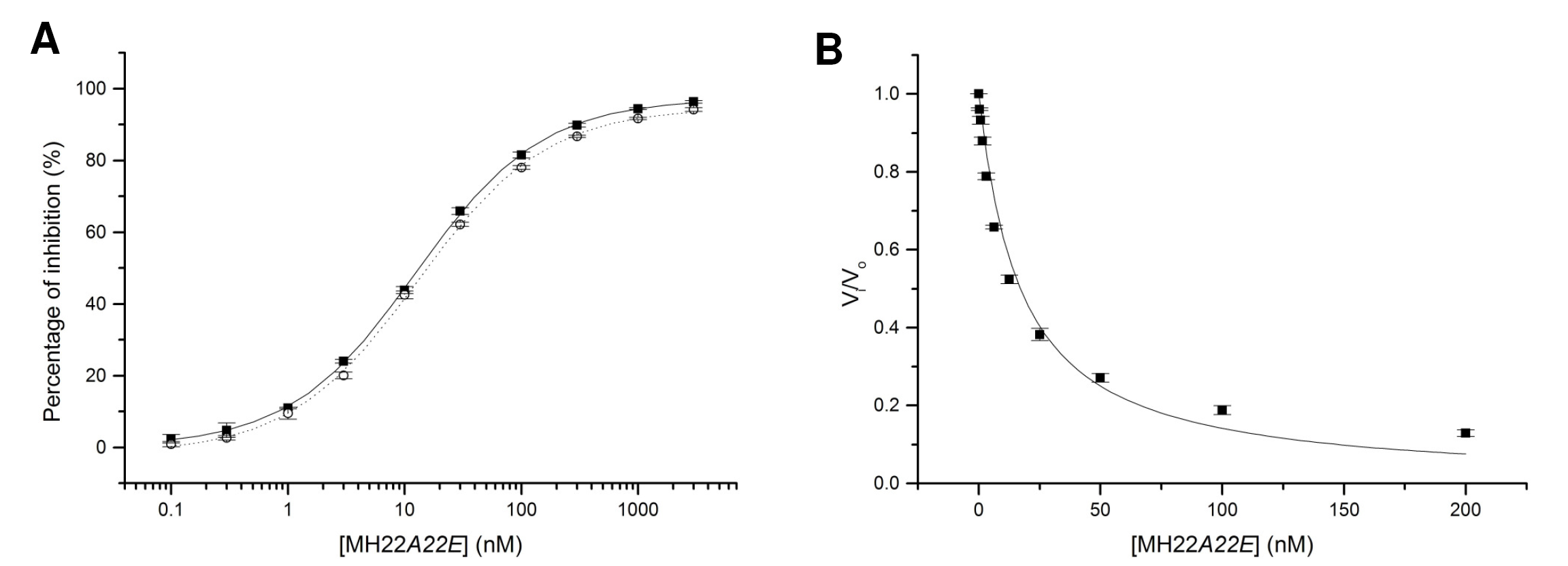

Supplement: Figure S14 — Variegin variant MH22 A22E (fast, tight-binding, noncompetitive inhibitor). (A) Dose-response curves of thrombin (1.65 nM) inhibited by MH22A22E (0.1 nM, 0.3 nM, 1 nM, 3 nM, 10 nM, 30 nM, 100 nM, 300 nM, 1000 nM and 3000 nM) in S2238 (100 µM) are independent of pre-incubation time. IC50 are 13.62±0.45 nM without pre-incubation (▪ solid line) and 15.6±0.4 nM with 20 min pre-incubation (○ dotted line) (n = 3, error bars represent S.D.). (B) Thrombin (1.65 nM) inhibition was tested with MH22A22E (0.39 nM, 0.78 nM, 1.56 nM, 3.13 nM, 6.25 nM, 12.5 nM, 25 nM, 50 nM, 100 nM and 200 nM) in S2238 (100 µM) (▪ solid line). Apparent inhibition constant Ki′ obtained by fitting data to equation (2), describing fast and tight-binding inhibitor, is 15.1±1.0 nM. Ki calculated from equations (4) and (5), describing noncompetitive inhibitors, is 15.1±1.0 nM (n = 3, error bars represent S.D.). (TIF) [file pone.0026367.s014.tif]

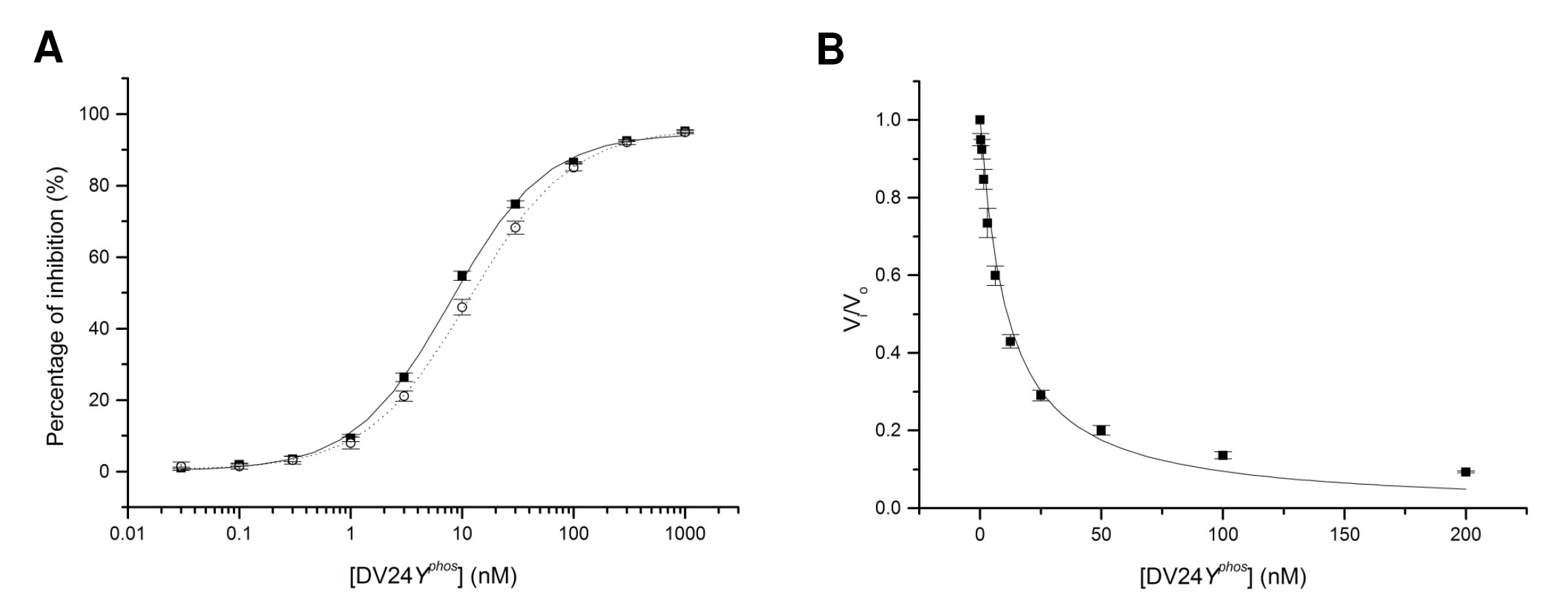

Supplement: Figure S15 — Variegin variant DV24 Yphos (fast, tight-binding, competitive inhibitor). (A) Dose-response curves of thrombin (1.65 nM) inhibited by DV24Yphos (0.03 nM, 0.1 nM, 0.3 nM, 1 nM, 3 nM, 10 nM, 30 nM, 100 nM, 300 nM and 1000 nM) in S2238 (100 µM) showed a right shift with increased pre-incubation time due to cleavage. IC50 are 8.67±0.45 nM without pre-incubation (▪ solid line) and 12.4±1.2 nM with 20 min pre-incubation (○ dotted line) (n = 3, error bars represent S.D.). (B) Thrombin (1.65 nM) inhibition was tested with DV24Yphos (0.39 nM, 0.78 nM, 1.56 nM, 3.13 nM, 6.25 nM, 12.5 nM, 25 nM, 50 nM, 100 nM and 200 nM) in S2238 (100 µM) (▪ solid line). Apparent inhibition constant Ki′ obtained by fitting data to equation (2), describing fast and tight-binding inhibitors, is 10.4±1.0 nM. Ki calculated from equation (3), describing competitive inhibitors, the inhibition constant is 0.327±0.032 nM (n = 3, error bars represent S.D.). (TIF) [file pone.0026367.s015.tif]

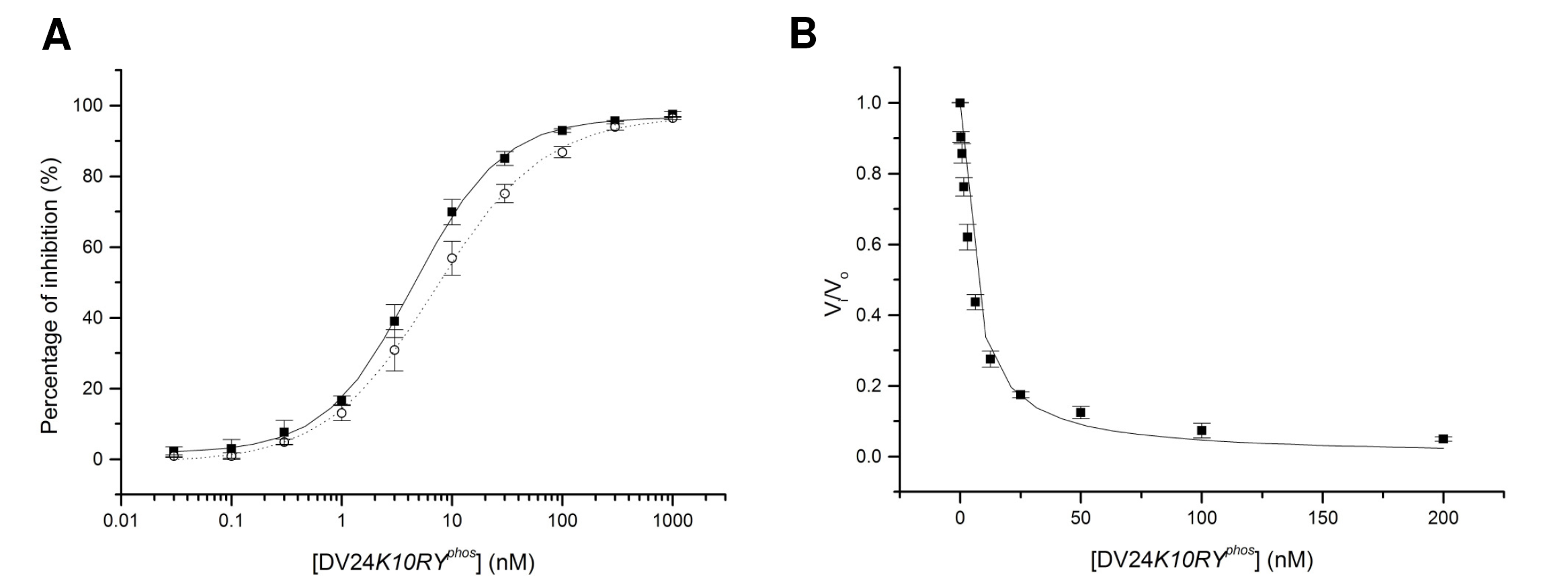

Supplement: Figure S16 — Variegin variant DV24 K10RYphos (fast, tight-binding, competitive inhibitor). (A) Dose-response curves of thrombin (1.65 nM) inhibited by DV24K10RYphos (0.03 nM, 0.1 nM, 0.3 nM, 1 nM, 3 nM, 10 nM, 30 nM, 100 nM, 300 nM and 1000 nM) in S2238 (100 µM) showed a right shift with increased pre-incubation time due to cleavage. IC50 are 4.64±0.78 nM without pre-incubation (▪ solid line) and 7.80±1.80 nM with 20 min pre-incubation (○ dotted line) (n = 3, error bars represent S.D.). (B) Thrombin (1.65 nM) inhibition was tested with DV24K10RYphos (0.39 nM, 0.78 nM, 1.56 nM, 3.13 nM, 6.25 nM, 12.5 nM, 25 nM, 50 nM, 100 nM and 200 nM) in S2238 (100 µM) (▪ solid line). Apparent inhibition constant Ki′ obtained by fitting data to equation (2), describing fast and tight-binding inhibitors, is 4.78±0.57 nM. Ki calculated from equation (3), describing competitive inhibitors, is 0.150±0.018 nM (n = 3, error bars represent S.D.). (TIF) [file pone.0026367.s016.tif]

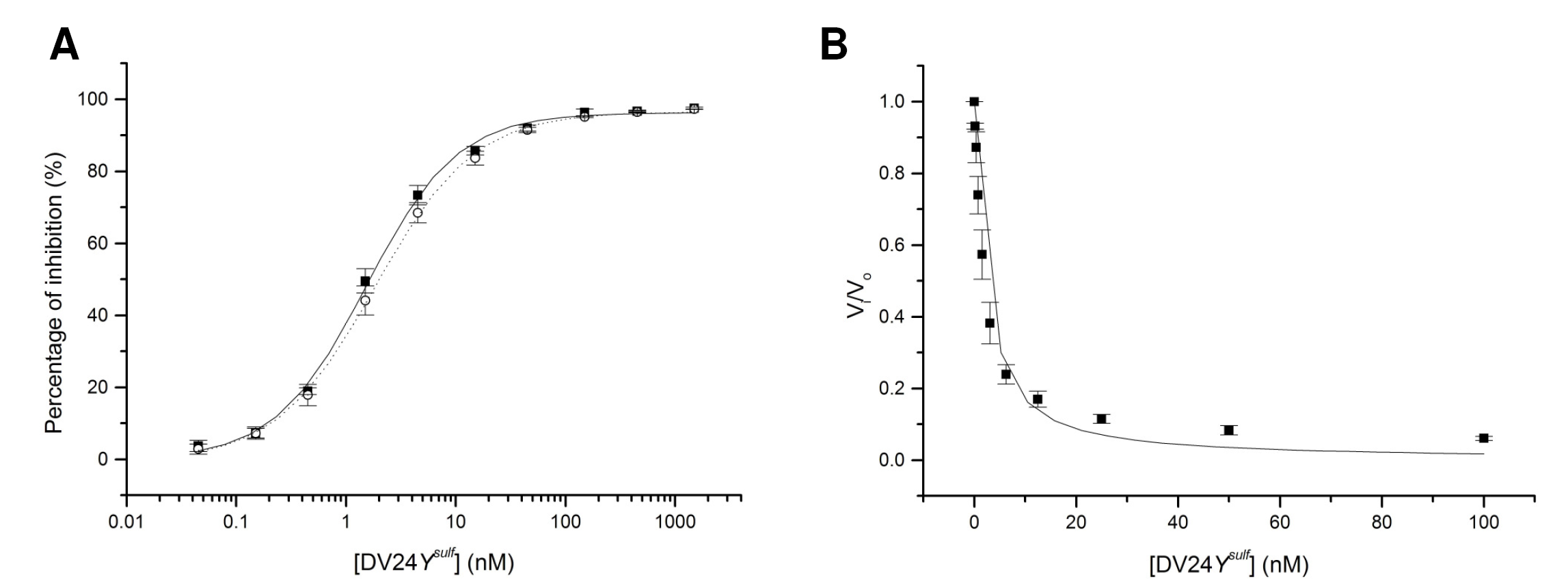

Supplement: Figure S17 — Variegin variant DV24 Ysulf (fast, tight-binding, competitive inhibitor). (A) Dose-response curves of thrombin (1.65 nM) inhibited by DV24Ysulf (0.05 nM, 0.15 nM, 0.45 nM, 1.5 nM, 4.5 nM, 15 nM, 45 nM, 150 nM, 450 nM and 1500 nM) in S2238 (100 µM) showed a right shift with increased pre-incubation time due to cleavage. IC50 are 1.66±0.18 nM without pre-incubation (▪ solid line) and 2.02±0.29 nM with 20 min pre-incubation (○ dotted line) (n = 3, error bars represent S.D.). (B) Thrombin (1.65 nM) inhibition was tested with DV24Ysulf (0.20 nM, 0.39 nM, 0.78 nM, 1.56 nM, 3.13 nM, 6.25 nM, 12.5 nM, 25 nM, 50 nM and 100 nM) in S2238 (100 µM) (▪ solid line). Apparent inhibition constant Ki′ obtained by fitting data to equation (2), describing fast and tight-binding inhibitor, is 1.78±0.47 nM. Ki calculated from equation (3), describing competitive inhibitors, is 0.056±0.015 nM (n = 3, error bars represent S.D.). (TIF) [file pone.0026367.s017.tif]

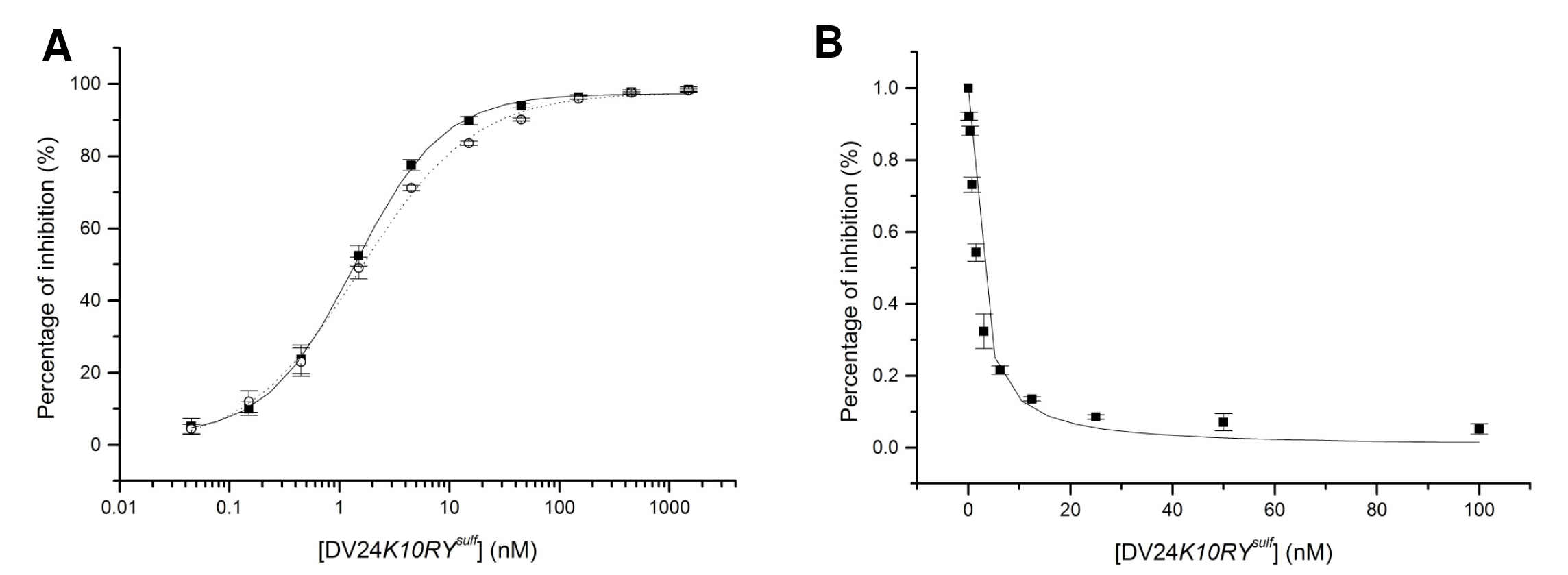

Supplement: Figure S18 — Variegin variant DV24 K10RYsulf (fast, tight-binding, competitive inhibitor). (A) Dose-response curves of thrombin (1.65 nM) inhibited by DV24K10RYsulf (0.05 nM, 0.15 nM, 0.45 nM, 1.5 nM, 4.5 nM, 15 nM, 45 nM, 150 nM, 450 nM and 1500 nM) in S2238 (100 µM) showed a right shift with increased pre-incubation time due to cleavage. IC50 are 1.39±0.17 nM without pre-incubation (▪ solid line) and 1.66±0.21 nM with 20 min pre-incubation (○ dotted line) (n = 3, error bars represent S.D.). (B) Thrombin (1.65 nM) inhibition was tested with DV24K10RYsulf (0.20 nM, 0.39 nM, 0.78 nM, 1.56 nM, 3.13 nM, 6.25 nM, 12.5 nM, 25 nM, 50 nM and 100 nM) in S2238 (100 µM) (▪ solid line). Apparent inhibition constant Ki′ obtained by fitting data to equation (2), describing fast and tight-binding inhibitors, is 1.33±0.19 nM. Ki calculated from equation (3), describing competitive inhibitors, is 0.0420±0.0061 nM (n = 3, error bars represent S.D.). (TIF) [file pone.0026367.s018.tif]

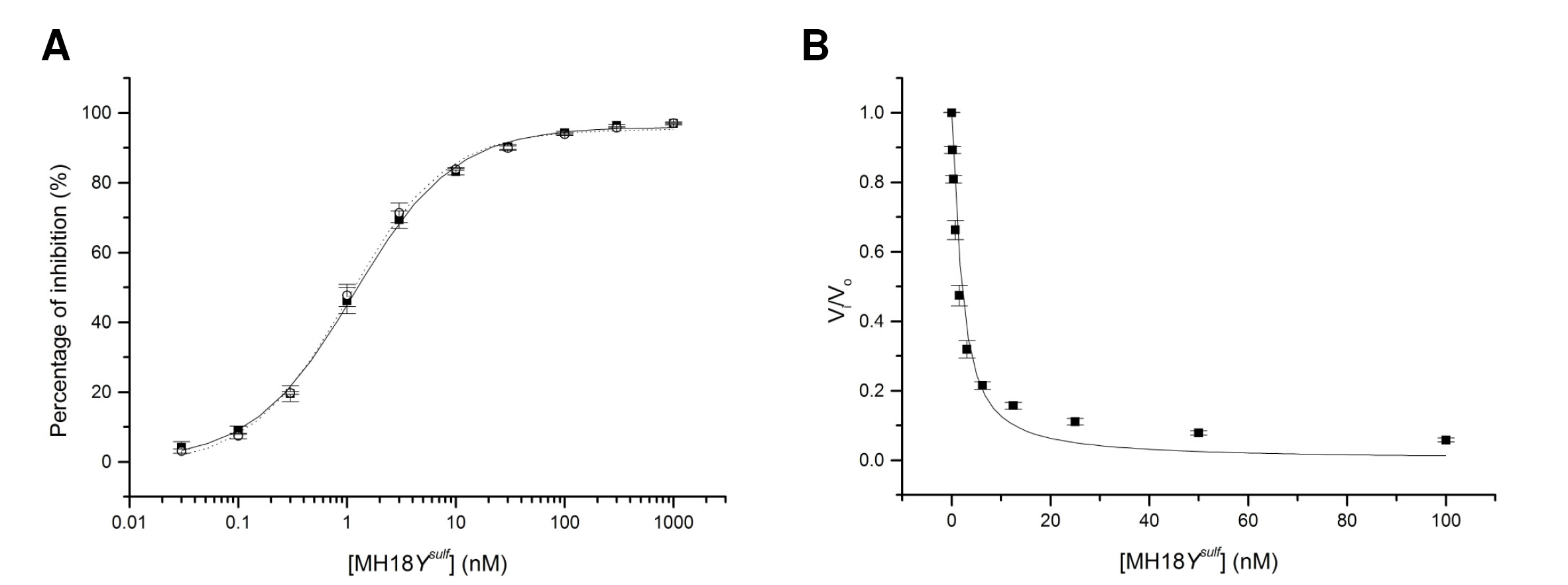

Supplement: Figure S19 — Variegin variant MH18 Ysulf (fast, tight-binding, noncompetitive inhibitor). (A) Dose-response curves of thrombin (1.65 nM) inhibited by MH18Ysulf (0.03 nM, 0.1 nM, 0.3 nM, 1 nM, 3 nM, 10 nM, 30 nM, 100 nM, 300 nM and 1000 nM) in S2238 (100 µM) are independent of pre-incubation time. IC50 are 1.26±0.18 nM without pre-incubation (▪ solid line) and 1.17±0.14 nM with 20 min pre-incubation (○ dotted line) (n = 3, error bar represents S.D.). (B) Thrombin (1.65 nM) inhibition was tested with MH18Ysulf (0.20 nM, 0.39 nM, 0.78 nM, 1.56 nM, 3.13 nM, 6.25 nM, 12.5 nM, 25 nM, 50 nM and 100 nM) in S2238 (100 µM) (▪ solid line). Apparent inhibition constant Ki′ obtained by fitting data to equation (2), describing fast and tight-binding inhibitors, is 1.25±0.18 nM. Ki calculated from equations (4) and (5), describing noncompetitive inhibitors, is 1.25±0.18 nM (n = 3, error bar represents S.D.). (TIF) [file pone.0026367.s019.tif]

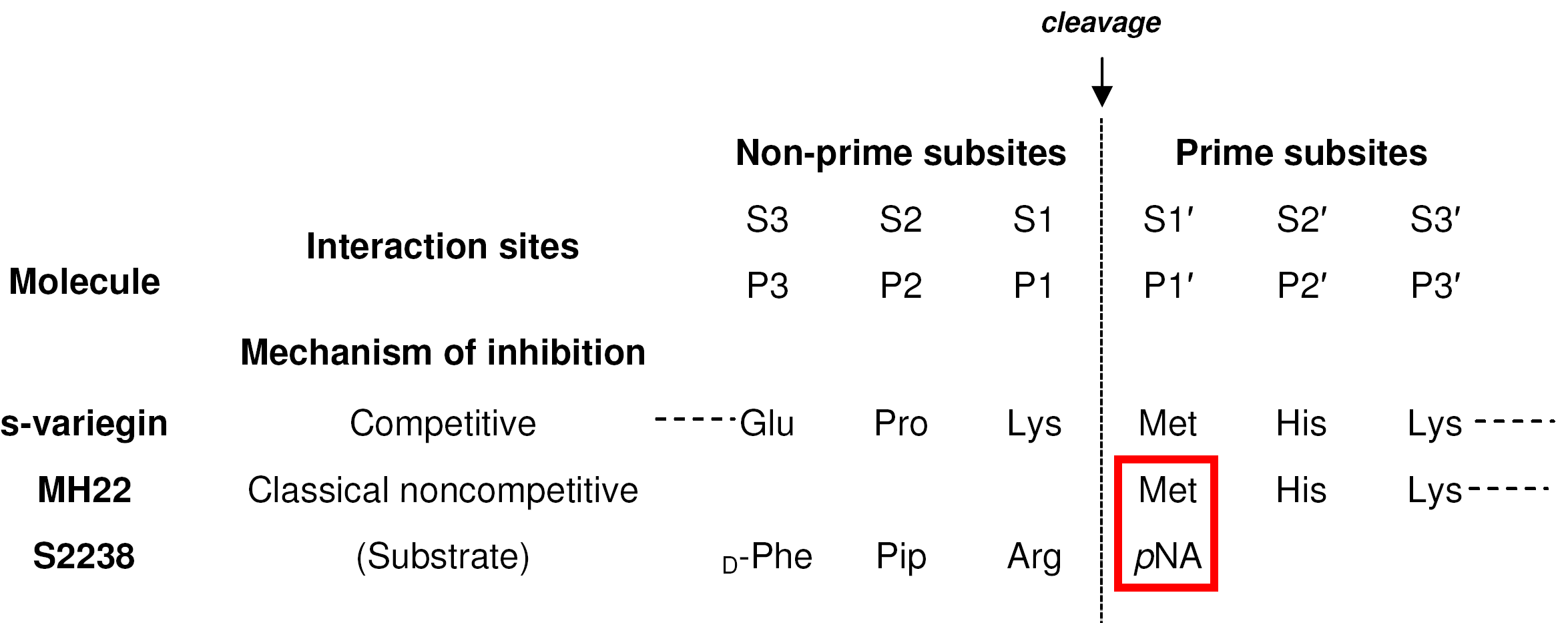

Supplement: Figure S20 — Noncompetitive inhibition of thrombin by MH22. s-Variegin binds to both the non-prime and prime subsites of thrombin active site and is cleaved between Lys-Met. After cleavage, the fragment C-terminal to the scissile bond (MH22) noncompetitively inhibits thrombin. The chromogenic substrate S2238 binds mainly to the non-prime subsites and is cleaved between Arg and para-nitroaniline (pNA). The overlaps between s-variegin and S2238 binding sites resulted in the observed competitive inhibition. In contrast, the noncompetitive inhibition observed for MH22 showed the lack of overlaps between MH22 and S2238 even in the S1′ subsite (red box). Indeed, no density was observed for P1′ Met in the present structure, most likely reflects the lack of contact with thrombin and hence leaves a free S1′ site for the binding of pNA moiety when MH22 is bound to thrombin. (TIF) [file pone.0026367.s020.tif]

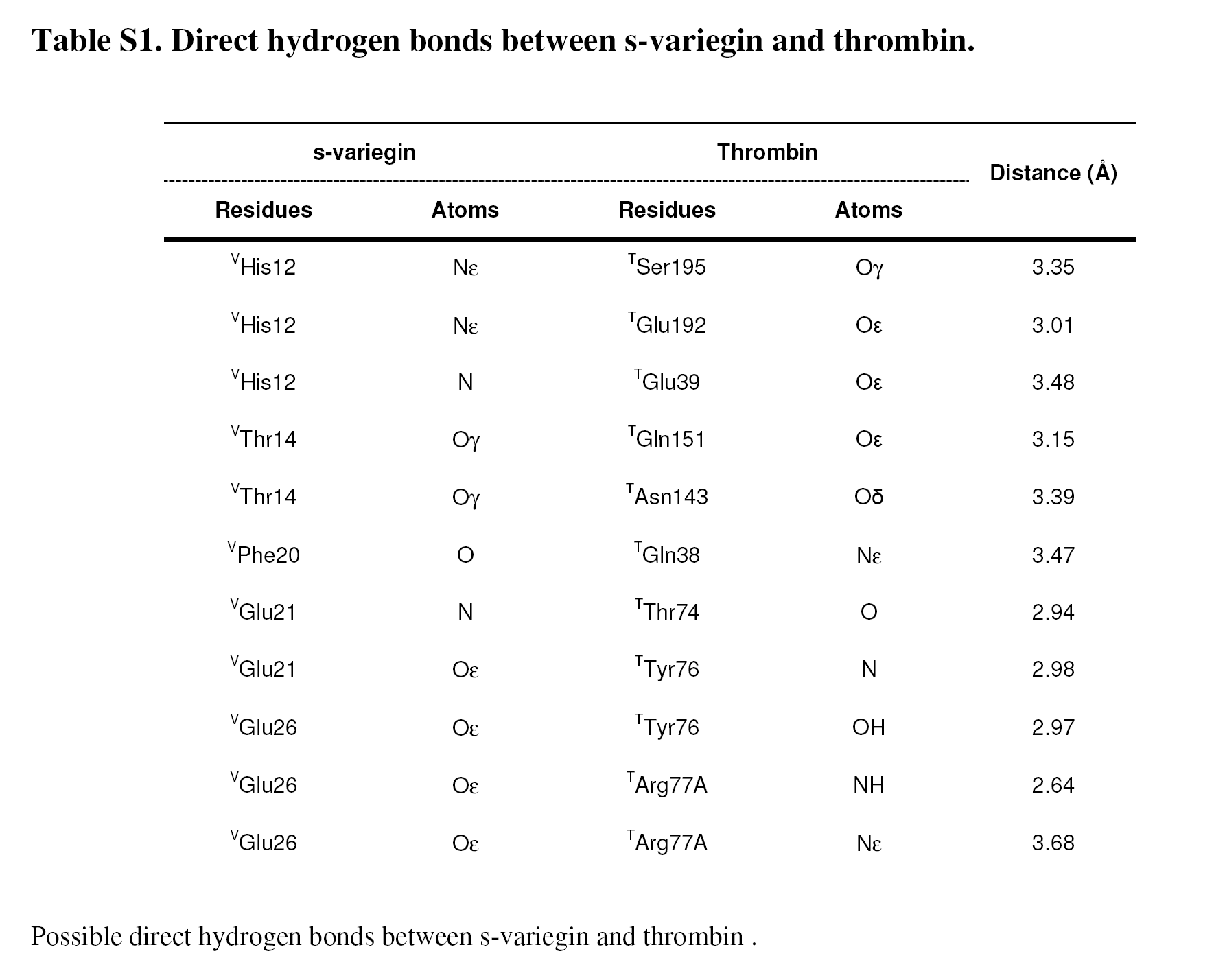

Supplement: Table S1 — A list of possible direct hydrogen bonds between s-variegin and thrombin calculated based on the online server PISA [28]. (TIF) [file pone.0026367.s021.tif]
