# Supplementary material for: Crystal Structure of Thrombin in Complex with S-Variegin: Insights of a Novel Mechanism of Inhibition and Design of Tunable Thrombin Inhibitors
Source: PLoS One. 2011 Oct 28;6(10):e26367. doi: 10.1371/journal.pone.0026367 (PMC3203879; doi:10.1371/journal.pone.0026367)
Supplement: Materials and Methods S1 — A detailed account for the selection and use of equations to fit the data of thrombin inhibitions is available in Materials and Methods S1. (DOC) [file pone.0026367.s022.doc]

**Materials and Methods S1**

**Materials**

Standard 9-Fluorenylmethyloxycarbonyl (Fmoc)-L-amino acids and Fmoc-PEG-PS (4-hydroxymethylphenoxyacetic acid linker) support resin were from Applied Biosystems (Foster City, CA, USA). Trifluoroacetic acid, formic acid and bovine serum albumin (BSA) were from Sigma Aldrich (St. Louis, MO, USA). Tyrosine sulfate derivative Fmoc-L-Tyr(SO_3_NnBu_4_)-OH and protamine sulfate were from Merck KGaA (Darmstadt, Germany). Human recombinant α-thrombin and human plasma derived thrombin were gifts from the Chemo-Sero-Therapeutic Research Institute (KAKETSUKEN, Japan)[1,2]. Chromogenic substrate *H*-_D_-Phe-pipecolyl-Arg-*p*NA•2HCl (S2238) were from Chromogenix (Milano, Italy).

**Thrombin**

Both recombinant human α-thrombin (used in crystallization and cleavage analysis of peptides) and human plasma derived thrombin (used in inhibition assays), were generous gifts from the Chemo-Sero-Therapeutic Research Institute (KAKETSUKEN, Japan). Peptides generally showed 2 fold stronger inhibitions against recombinant α-thrombin than plasma derived thrombin (reason unknown).

**Michaelis-Menten constant (*K_m_*) of S2238 for thrombin**

Assays for thrombin amidolytic activity were carried out using the small, synthetic chromogenic substrate S2238. Hydrolysis of S2238 released colored product *p*-nitroaniline (*p*NA). Rate of *p*NA formation is proportional to the enzymatic activity and followed at 405 nm. The relation between the initial rates of reaction, *V*, with concentration of S2238 follows Michaelis-Menten kinetics.

Assays were performed in 96-well microtiter plates in 50 mM Tris buffer (pH 7.4) containing 100 mM NaCl and 1 mg/ml BSA at room temperature. The rates of formation of colored product pNA were followed at 405 nm for 10 min with a SPECTRAMax Plus microplate spectrophotometer (Molecular Devices, Sunnyvale, CA, USA). Data obtained were fitted to equation **(1)** using Origin software (MicroCal, Northampton, MA, USA) to calculate *K_m_* and *V_max_*. *K_m_* of S2238 for human plasma derived thrombin is 3.32 ± 0.35 μM and *V_max_* is 50.5 ± 2.2 mOD/min, similar to the value obtained using recombinant human α-thrombin (*K_m_* = 3.25 ± 0.56 μM, *V_max_* = 39.5 ± 2.0 mOD/min) and other reported values in the literature[3,4].

**Inhibition of thrombin amidolytic activity**

The activity of each peptide was determined by the inhibition of thrombin amidolytic activity assayed as described above. The rate of increase in absorbance in the absence of inhibitor was considered as 0% inhibition. Dose-response curves were fitted using Origin software to calculate *IC_50_* value (the concentration needed to reduce the thrombin amidolytic activity by 50 %) with the following logistic sigmoidal equation:

*y* = *A_2_* + (*A_1_* - *A_2_*) / [1 + (*x* / *x_0_*)*^H^*] **(1)**

where *y* is percentage of inhibition, *A_2_* is right horizontal asymptote, *A_1_* is left horizontal asymptote, *x* is log_10_ of inhibitor concentration, *x_0_* is point of inflection and *H* is the slope of the curve. *IC_50_* was calculated by substituting ‘50’ into *y*.

To determine the inhibition constant, *K_i_*, of each peptide, the following equations were considered: When an enzyme is inhibited by an equimolar concentration of inhibitor, the binding of inhibitor to enzyme causes a significant depletion in the concentration of free inhibitors. This tight-binding inhibition is described by equation **(2)**[4]:

*v_s_* = (*v_o_*/2*E_t_*) {[(*K_i_*’ + *I_t_* – *E_t_*)^2^ + 4*K_i_’E_t_*]^1/2^ – (*K_i_’* + *I_t_* – *E_t_*)} **(2)**

where *v_s_* is steady state velocity in the presence of inhibitor, *v_o_* is velocity observed in the absence of inhibitor, *E_t_* is total enzyme concentration, *I_t_* is total inhibitor concentration and *K_i_’* is apparent inhibition constant.

For competitive inhibition, *K_i_* is related to *K_i_’* by equation **(3)**:

*K_i_’* = *K_i_* (1 + *S*/*K_m_*) **(3)**

where *K_i_’* increases linearly with *S*, *K_i_* is the inhibition constant, *S* is the concentration of substrate and *K_m_* is the Michaelis-Menten constant for S2238.

For noncompetitive inhibitors, *K_i_* is related to *K_i_’* by equation **(4)**[5]:

*K_i_’* = (*S* + *K_m_*) / [(*K_m_*/*K_i_*) + (*S*/*αK_i_*)] **(4)**

where *α* is the modifying constant of the inhibitor on the affinity of the enzyme for its substrate, and likewise the effect of the substrate on the affinity of the enzyme for the inhibitor. *α* < 1 when binding of one supported the other, *α* > 1 when binding of one impedes the other and when *α* = 1, binding of one has no effect on the other. For a mixed-type noncompetitive inhibitor, *α* is either < 1 or > 1. For classical noncompetitive inhibitors, *α* = 1, thus,

*K_i_’* = *K_i_* **(5)**

where *K_i_’* remained constant with increasing *S*, *K_i_* is the inhibition constant, *S* is the concentration of substrate S2238 and *K_m_* is the Michaelis-Menten constant for S2238.

For peptides that were found to be tight-binding inhibitors, the data were fitted to these equations using Origin software.

If the rate of interaction between the inhibitor and the enzyme is slow so that the inhibited steady-state velocity is slowly achieved, the progress curve of product formation of this slow binding inhibition is described by equation **(6)**[6]:

*P* = *v_f_t* + (*v_i_* – *v_f_*) (1 – e^-^*^kt^*) / *k* + *P_o_* **(6)**

where *P* is the amount of product formed, *P_o_* the is initial amount of product, *v_f_* is final steady state velocity, *v_i_* is initial velocity, *t* is time, and *k* is apparent first-order rate constant.

The simplest kinetic mechanism to describe variegin peptides that exhibit slow binding property follows scheme 1[6,7]:

E + I EI EI* **Scheme (1)**

*k_1_*

*k_2_*

*k_3_*

*k_4_*

where EI is initial collision complex, *k_3_* is forward isomerization rate and *k_4_* is reverse isomerization rate. In this scheme, binding involves rapid formation of an initial collision complex (EI) that subsequently undergoes slow isomerization to the final enzyme-inhibitor complex (EI*). *k* increases hyperbolically with inhibitor concentrations. Dissociation constant of EI (denoted *K_i_`*) can be calculated from equation **(7)**:

*k* = *k_4_* + *k_3_I_t_* / [*I_t_* + *K_i_`*(1 + *S* / *K_m_*)] **(7)**

The overall inhibition constant *K_i_* can be calculated from equation **(8)**:

*K_i_* = *K_i_`* [*k_4_* / (*k_3_* + *k_4_*)] **(8)**

For peptides that were found to be a slow binding inhibitor their data were fitted to these equations using Origin software. Due to the low initial activity of slow binding inhibitors, the concentrations of inhibitors used in kinetic assays are at least 11 fold in excess of thrombin. Therefore, the ‘tight-binding’ consideration (where binding to enzymes cause significant depletion of the amount of free inhibitors) for slow binding inhibitors presented here does not apply.

References

1. Yonemura H, Imamura T, Soejima K, Nakahara Y, Morikawa W, Ushio Y, Kamachi Y, Nakatake H, Sugawara K, Nakagaki T, Nozaki C (2004) Preparation of recombinant alpha-thrombin: high-level expression of recombinant human prethrombin-2 and its activation by recombinant ecarin. J Biochem (Tokyo) 135: 577-582.

2. Soejima K, Mimura N, Yonemura H, Nakatake H, Imamura T, Nozaki C (2001) An efficient refolding method for the preparation of recombinant human prethrombin-2 and characterization of the recombinant-derived alpha-thrombin. J Biochem (Tokyo) 130: 269-277.

3. Myles T, Le Bonniec BF, Betz A, Stone SR (2001) Electrostatic steering and ionic tethering in the formation of thrombin-hirudin complexes: the role of the thrombin anion-binding exosite-I. Biochemistry 40: 4972-4979.

4. Stone SR, Hofsteenge J (1986) Kinetics of the inhibition of thrombin by hirudin. Biochemistry 25: 4622-4628.

5. Copeland, R. A. (2000) Enzymes: a practical introduction to structure, mechanism and data analysis. Wiley-VCH, New York.

6. Morrison JF, Walsh CT (1988) The behavior and significance of slow-binding enzyme inhibitors. Adv Enzymol Relat Areas Mol Biol 61: 201-301.

7. Rezaie AR (2004) Kinetics of factor Xa inhibition by recombinant tick anticoagulant peptide: both active site and exosite interactions are required for a slow- and tight-binding inhibition mechanism. Biochemistry 43: 3368-3375.
